# Nanodiamond autophagy inhibitor allosterically improves the arsenical-based therapy of solid tumors

**DOI:** 10.1038/s41467-018-06749-2

**Published:** 2018-10-19

**Authors:** Zhifen Cui, Yu Zhang, Kai Xia, Qinglong Yan, Huating Kong, Jichao Zhang, Xiaolei Zuo, Jiye Shi, Lihua Wang, Ying Zhu, Chunhai Fan

**Affiliations:** 1 Division of Physical Biology and Bioimaging Center, Shanghai Synchrotron Radiation Facility, Shanghai Institute of Applied Physics, Chinese Academy of Sciences, University of Chinese Academy of Sciences, Shanghai, 201800 China; 2 School of Chemistry and Chemical Engineering, Institute of Molecular Medicine, Renji Hospital, School of Medicine, Shanghai Jiao Tong University, Shanghai, 200240 China; 3 Kellogg College, University of Oxford, Banbury Road, Oxford, OX2 6PN UK; 4 Shanghai Key Laboratory of Green Chemistry and Chemical Processes, School of Chemistry and Molecular Engineering, East China Normal University, 500 Dongchuan Road, Shanghai, 200241 China

## Abstract

Arsenic trioxide (ATO) is a successful chemotherapeutic drug for blood cancers via selective induction of apoptosis; however its efficacy in solid tumors is limited. Here we repurpose nanodiamonds (NDs) as a safe and potent autophagic inhibitor to allosterically improve the therapeutic efficacy of ATO-based treatment in solid tumors. We find that NDs and ATO are physically separate and functionally target different cellular pathways (autophagy vs. apoptosis); whereas their metabolic coupling in human liver carcinoma cells remarkably enhances programmed cell death. Combination therapy in liver tumor mice model results in ~91% carcinoma decrease as compared with ~28% without NDs. Treated mice show 100% survival rate in 150 days with greatly reduced advanced liver carcinoma-associated symptoms, and ~80% of post-therapy mice survive for over 20 weeks. Our work presents a novel strategy to harness the power of nanoparticles to broaden the scope of ATO-based therapy and more generally to fight solid tumors.

## Abstract (web summary)

Arsenic trioxide (ATO) based therapy in solid cancers is limited. Here they repurpose nanodiamonds (NDs) as a safe and potent autophagic inhibitor to improve the efficacy of ATO-based treatment in solid tumors and show the combination therapy to work better in orthotopic liver cancer model.

## Introduction

Arsenic trioxide (ATO)-based cancer therapy has attracted intense interest since low concentrations of ATO can selectively induce apoptosis of blood cancer cells^1–3^. Especially, the complete remission (CR) rate of arsenical-based therapy has reached ~95% in patients with acute promyelocytic leukemia (APL), making it become the first cured leukemia^1–3^. Nevertheless, the extraordinary success of ATO in curing blood cancers is not effectively replicated in treating solid tumors^4,5^. Previous studies suggested that the resistance to programmed cell death might arise from autophagic induction of ATO in various solid tumor cells^6^.

Macroautophagy (hereafter called autophagy) is a conserved catabolic process that maintains cellular homeostasis by recycling proteins or cell organelles. Due to the tight relationship between autophagy and metabolic fitness pathways of tumor cells^7–13^, autophagy is often activated in response to a variety of chemotherapeutic treatments of solid tumors^14,15^, which may rescue the drug-induced apoptosis and enable continuous survival of cancer cells^16^. Given that, disruption of the crosstalk between autophagy and apoptosis holds high therapeutic potential^10,11,13,17,18^ especially for ATO-based treatment of solid tumors. Current clinical efforts to inhibit autophagy are focused on chemical drugs including chloroquine (CQ) or hydroxychloroquine (HCQ). However, these chemical inhibitors are often associated with various side effects^19–21^. Additionally, the acidic pH in the tumor tissue makes it difficult for CQ to block autophagy^22^.

In this work, we aim to explore the use of nanoparticle autophagy inhibitors (NAPI) for enhanced ATO therapy for solid tumors. Nanomedicine holds great promise for cancer therapy arising from diverse properties of nanoparticles for controlled delivery, enhanced intracellular tracking, and intelligent response^23–25^. Evidence has also accumulated that various types of nanoparticles (NPs) can modulate autophagic responses in a number of mammalian cell lines and in vivo^26–29^. Here, by screening a library of NAPIs in human liver carcinoma (HepG2) cells, we establish that nanodiamonds (NDs) are a type of safe and potent NAPI, which can allosterically enhance the ATO-based therapy in HepG2. Based on this finding, we develop a combination therapy for ATO-based treatment of an orthotopic liver tumor transplantation mice model.

## Results

### Screening of potent NAPIs in HepG2

**Fig. 1:**
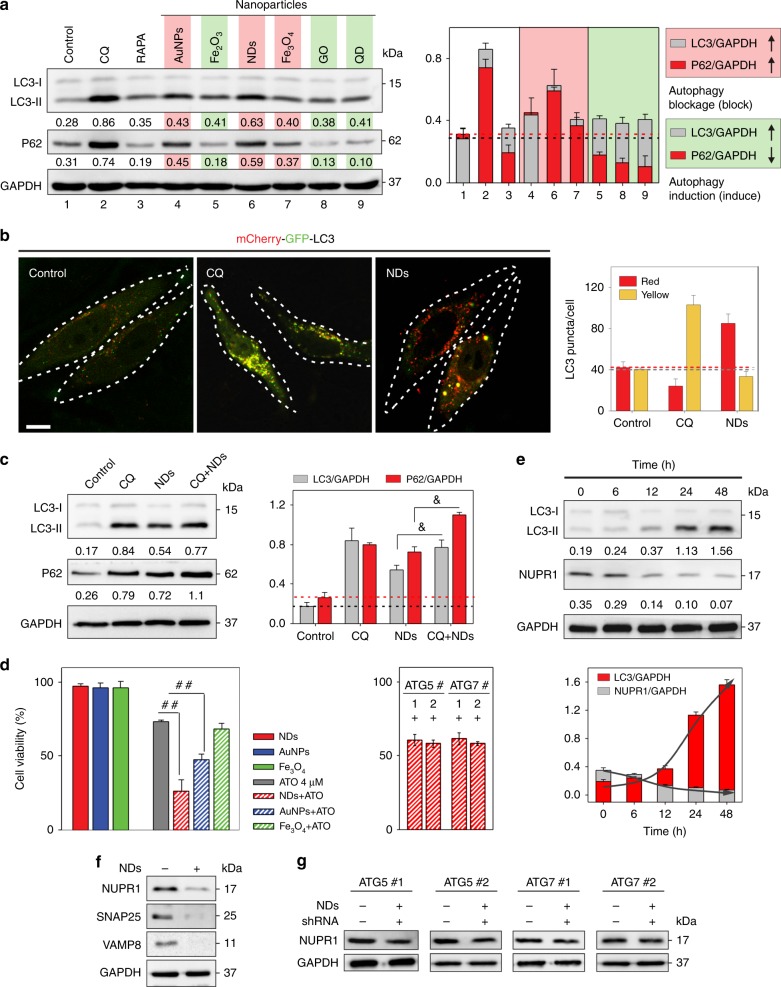
Screening of potent NAPIs in HepG2. **a** Immunoblots for autophagy-related proteins LC3-II, p62 (left); semi-quantified analysis (*n* = 3) in various nanoparticles treated cells (right). CQ and Rapamycin (Rapa) were used as positive controls for autophagy inhibition and autophagy activation, respectively. Glyceraldehyde 3-phosphate dehydrogenase (GAPDH) was used as the loading control. Normalized band densities were shown below each band. **b** Fluorescence images of mCherry-GFP-LC3 cells after incubation with CQ or NDs for 48 h (autophagosomes: mCherry+/GFP+ yellow puncta; autolysosomes: mCherry+/GFP) and quantification of the number of LC3 puncta per cell in cells (10 cells per group). Scale bar: 10 μm. **c** Immunoblots for autophagy-related proteins LC3-II, p62 (left); semi-quantified analysis (*n* = 3) in CQ, NDs, or CQ–NDs-treated cells (right). ^&^*P* < 0.05, significantly different from NDs. GAPDH was used as the loading control. Normalized band densities were shown below each band. **d** Left: Cell viability after incubation with ATO or various NAPIs–ATO mixture for 48 h (*n* = 3). ^##^*P* < 0.01 by *t*-test, significantly different from ATO. Right: Cell viability after 48 h NDs–ATO treatment with RNAi of autophagy proteins ATG5 and ATG7 (*n* = 3). **e**, **f** Immunoblots for autophagy-related protein LC3-II and autolysosomal process-related protein NUPR1, SNAP25, VAMP8 in NDs-treated cells. **g** Immunoblots for autolysosomal process-related protein NUPR1 after NDs treatment with RNAi of autophagy proteins ATG5 and ATG7. GAPDH was used as the loading control. Error bars are s.d.

Various NPs have proven to affect the cell autophagy process at different levels^26^. To identify a safe and potent NAPI, we first tested the cellular effects of a series of NPs on HepG2, including metal (Au), metal oxide (Fe_2_O_3_ and Fe_3_O_4_), carbon (NDs and graphene oxide, GO), and semiconductor (CdSe quantum dot, QD) NPs. We found that NDs, AuNPs, and Fe_3_O_4_ NPs increased accumulation of phosphatidylethanolamine-conjugated LC3 (named LC3-II) and an autophagy substrate p62^30^, with NDs the strongest ones; whereas other NPs increased processing of LC3 conversion and degradation of p62 (Fig. 1a and Supplementary Fig. 1). To further assess the autophagic role of NPs in cells, we performed genetic interference experiments by transfecting HepG2 cells with small hairpin RNAs (shRNAs) targeting ATG5 or ATG7, which restrains autophagy initiation^31^. The efficiency of shRNAs in autophagy deregulation was assessed with western blotting (Supplementary Fig. 2). We found that ATG5/7 depletion significantly mitigated LC3 conversion induced by NPs, which confirmed the NP-induced autophagy inhibition/induction (Supplementary Fig. 3). In NDs-treated cells, TEM imaging revealed the presence of a large amount of vesicles in HepG2 cells, with encapsulated NDs (Supplementary Fig. 4). These vesicles typically contain electron-dense cytoplasmic remnants, consistent with the features of degradative structures within autolysosomes. We further employed a tandem reporter construct, mCherry-GFP-LC3^31^, to differentiate the mechanism for the dysfunction in autolysosomal processing. We found that CQ treatment led to an increase of yellow-color-labeled LC3 puncta (mCherry-GFP-LC3-positive autophagosomes), characteristic of the increase in autophagosome–lysosome flow^32^. However, the NDs treatment led to an increase of red-color-labeled LC3 puncta (mCherry-positive, GFP-fluorescence-negative autolysosomes) (Fig. 1b and Supplementary Fig. 5), suggesting the interference in autolysosomal clearance. Hence, NDs take a distinctly different approach to inhibit autophagy with the classic CQ. In the co-presence of CQ and NDs, we found that LC3-II increased and more p62 accumulated in cells, as compared to cells treated with NDs alone (Fig. 1c), which further confirmed that NDs functioned as autophagy inhibitors to regulate autophagic flux and autolysosomal efflux. Taken together, we substantiated the autophagy inhibition role of NDs in cells. We further revealed that the inhibition mechanism of NDs is distinctly different with CQ, and that the co-presence of the two could enhance the autophagic level.

We next explored whether these NPs could affect cell death of ATO-treated HepG2. We found that autophagy-induction NPs did not show significant effects on cell death (Supplementary Fig. 6). In contrast, both NDs and AuNPs increased the ATO-induced cell death, although the other NAPI Fe_3_O_4_ NPs had minimal effects (Fig. 1d, left). NDs appeared to be the most effective autophagic inhibitors in these NPs. Moreover, ATG5/7 depletion significantly mitigated NDs-mediated improvement of ATO-induced cell death (Fig. 1d, right). Of note, ATG5/7 depletion alone minimally interfered with the cell viability (Supplementary Fig. 7). Hence, this genetic strategy confirmed that the NDs responses observed in this work were directly related to the inhibition of the autophagic pathway.

Having established that NDs are a type of potent NAPI, we next explored mechanistic roles of NDs in the autophagy-lysosomal processing. We monitored the expression of NUPR1 (nuclear protein 1, transcriptional regulator) that regulates autophagic flux and autolysosomal clearance in cells^32^. We found that the NDs treatment led to decreased NUPR1 levels in a time-dependent manner, which is consistent with the observations that the LC3-I to LC3-II conversion increased over time (Fig. 1e). Hence, the NUPR1 deregulation is closely related to the autophagic flux change induce by NDs. Further mechanistic studies revealed that the NDs treatment did not lead to significant change of the processing of the lysosomal proteases cathepsins B/D into their fully active forms in cells, suggesting that defective lysosomal cathepsin processing was not the primary effect of NDs (Supplementary Fig. 8). In contrast, we found that the NDs treatment caused deregulation of SNAP25 and VAMP8 (Fig. 1f), which were previously found to be involved in the NUPR1-mediated autolysosomal efflux^32^. Moreover, ATG5/7 depletion significantly mitigated NUPR1 depletion induced by NDs (Fig. 1g), further confirming that NUPR1 depletion impairs autolysosomal efflux in NDs-treated cells. Taken together, we demonstrate that NDs function as autophagy inhibitors via impairing NUPR1-mediated autolysosomal efflux in cells.

### Mechanistic analysis of autophagic inhibition in ATO-treated HepG2

**Fig. 2:**
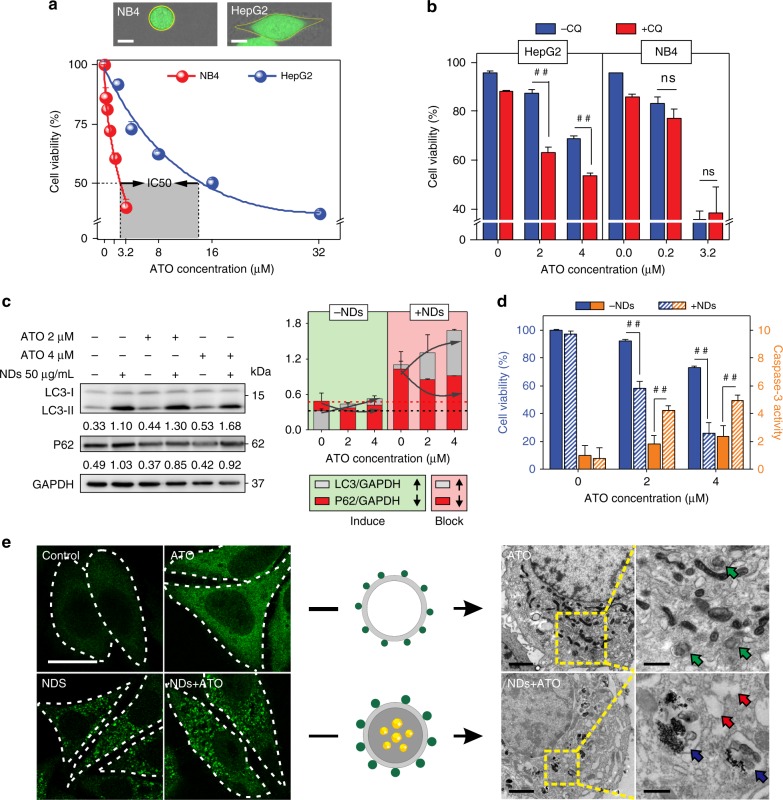
Mechanistic analysis of autophagic inhibition in ATO-treated HepG2. **a** The viability of HepG2 and NB4 cells after incubation with ATO at various concentrations for 48 h (*n* = 3; error bars are s.d.). Upper: Confocal images of NB4 or HepG2 cells after being stained with calcein AM. Scale bars: 10 μm. **b** The viability of HepG2 and NB4 cells after incubation with CQ, ATO, or CQ–ATO mixture for 48 h (*n* = 3). ^##^*P* < 0.01 by *t*-test, significantly different from ATO. ns not significant. **c** Immunoblots of autophagy-related proteins LC3-II, p62 (left); semi-quantified analysis (*n* = 3) in NDs, ATO, or NDs–ATO mixture-treated cells (right). GAPDH was used as the loading control. Normalized band densities were shown below each band. **d** Cell viability and Caspase-3 activity after incubation with ATO or NDs–ATO mixture for 48 h (*n* = 3). ^##^*P* < 0.01 by *t*-test, significantly different from ATO. **e** Immunostaining of LC3 in NDs, ATO, or NDs–ATO mixture-treated cells (left). Scale bar: 20 μm. TEM images of ATO or NDs–ATO mixture-treated cells (right). Scale bars: 1 μm. Typical structures of autophagosomes induced by ATO and autolysosome containing NDs induced by NDs–ATO mixture are indicated with green or blue arrows, respectively (magnification). Vacuoles inside cells are indicated with red arrows (magnification). Scale bars: 500 nm. Error bars are s.d.

Next, we investigated the molecular mechanism of autophagic inhibition in the enhanced cell death of ATO-treated HepG2. We first studied the therapeutic difference of ATO in HepG2 and human APL (NB4) cells, corresponding to the solid tumor and APL cell lines, respectively^33^. We found that the effective concentration of ATO for NB4 was one order of magnitude lower than that for HepG2 (Fig. 2a). Western blot analysis of autophagy-related protein expression (LC3-II and P62) revealed that ATO induced autophagy in HepG2 but not in NB4 (Supplementary Fig. 9), which might be responsible for their remarkable difference in sensitivity to the ATO treatment.

To establish the role of autophagic inhibition in increased cell death of ATO-treated HepG2, we employed the chemical inhibitor CQ to block ATO-induced autophagy. Western blot analysis showed that the addition of CQ (12.5 µg mL^−1^) further increased the ATO-induced accumulation of LC3-II, whereas potently blocked ATO-induced p62 degradation in HepG2 cells (Supplementary Fig. 9), confirming its blockage effect on ATO-induced autophagy. We also noted that the co-treatment of HepG2 with CQ and ATO enhanced the cytotoxicity similar to the effect of NDs. In contrast, co-treatment of NB4 with CQ and ATO did not show significantly increased cell death, suggesting that autophagy does not play a role in ATO-treated APL cells (Fig. 2b and Supplementary Fig. 10).

Having substantiated that autophagic inhibition plays an important role in ATO-based cytotoxicity in HepG2, we explored how NDs affected the ATO-treated HepG2. Similar to that of CQ, we observed the increased accumulation of LC3-II and blockage of p62 degradation in western blot, when NDs were added to ATO-treated HepG2 cells (Fig. 2c). Confocal microscopy further showed that co-treatment of NDs significantly reduced the formation of small LC3-positive dots that were induced by ATO, while increased the accumulation of LC3-positive punctuate structures in cells (Fig. 2e, left). TEM images revealed that co-treatment significantly reduced the ATO-induced autophagosome formation, and induced the accumulation of NDs-containing autolysosome (Fig. 2e, right). More importantly, after the co-treatment, we observed the presence of many vacuoles inside the cells (Fig. 2e, right), together with the increased caspase-3 activity and the decreased cell viability (Fig. 2d), suggesting the improvement of ATO-induced programmed cell death (apoptosis).

### NDs autophagy inhibitor improves the ATO-based therapy in vivo

**Fig. 3:**
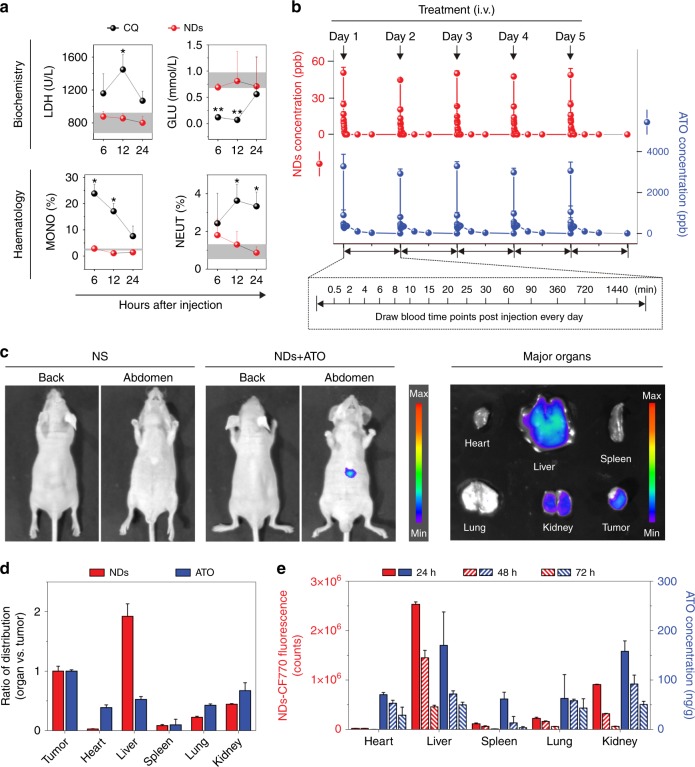
In vivo biocompatibility assessment of NDs/ATO co-treatment. **a** Nude mice were administered intravenously (i.v.) with normal saline (NS, 200 μL, *n* = 3), NDs (5 mg kg^−1^, *n* = 9) or CQ (10 mg kg^−1^, *n* = 9). Blood test results for treated mouse. The region rendered in gray represents the range observed in mouse treated with control NS (Supplementary Table 2). Error bars represent one standard deviation above or below the mean. *P < 0.05; **P < 0.01, significantly different from NS (*t*-test). **b**–**e** HepG2 tumor-bearing nude mice were administered i.v. with NDs–1.5 mg kg^−1^ ATO mixture daily for 5 consecutive days. **b** The blood circulation curve of NDs-CF770/ATO determined by measuring CF770 fluorescence/ATO concentration in the blood at different time points post-injection every day (*n* = 10). **c** Representative whole-body images of tumor-bearing nude mice (left) and their major organ images (right) at 48 h after the end of administration. The quantities indicated by the color bar ranged from 5500 to 10,700 cps in **c**, left and 700 to 3700 cps in **c**, right. **d** Distribution ratio of the NDs/ATO accumulated in the tumor vs. healthy tissues at 48 h after the end of administration (*n* = 3). **e** Quantification of NDs/ATO accumulated in mouse tissues at different time point after the end of administration (*n* = 3 at each time point). Error bars are s.d.

To assess the therapeutic potential of NDs in ATO-based therapy, we first performed a series of in vitro and in vivo biocompatibility tests to evaluate the therapeutic safety of NDs/ATO co-treatment. Cell-viability assay revealed that NDs did not exhibit significant cytotoxicity even in the high-concentration regime (100 μM). This compares favorably with CQ, a type of chemical inhibitor that has been in clinical trial^21^, which exhibited dose-dependent cytotoxicity (Supplementary Fig. 11). Standard hematological and biochemical markers in blood after administration of NDs or CQ revealed no obvious change after the NDs treatment in all parameters; whereas significant changes in several parameters after the CQ treatment at different time intervals (Fig. 3a and Supplementary Table 2). Our observations are also consistent with the previous reports that CQ (or HCQ) has various side effects^19,21^. We further studied the pharmacokinetics of NDs/ATO in vivo, NDs were labeled with a near-IR dye, XenoLight CF770^34^ (Supplementary Fig. 12), and ATO concentrations were determined via ICP-MS. We constructed an orthotopic tumor transplantation model that simulates the clinical features for liver cancer research. We observed that the blood circulation curve of NDs/ATO remained nearly constant every day and they were cleared from the blood within 1/24 h after each administration (Fig. 3b and Supplementary Fig. 13). Further, we monitored their in-vivo biodistribution and clearance. At 48 h after the end of administration, we observed fluorescent signals of NDs were predominantly in the tumor, liver, and kidney from whole-body and organ imaging (Fig. 3c). Quantitative analysis showed that NDs were also accumulated in spleen and lung. ATO was predominantly in the tumor, heart, liver, spleen, lung, and kidney at this timepoint (Fig. 3d). Most accumulated NDs were cleared in all organs after 72 h, which was right at the time when the second cycle started (Fig. 3e). Thus, we consider that NDs could be cleared from the body at the end of the treatment. More importantly, our analysis revealed that at 48 h after the end of administration (the last day of the first cycle), the relative amount of NDs/ATO in heart, liver, spleen, lung, and kidney to tumor site was 0.03/0.38, 1.92/0.53, 0.08/0.10, 0.22/0.43, and 0.44/0.67, respectively (Fig. 3d), which confirms the tumor-targeting ability of NDs/ATO.

**Fig. 4:**
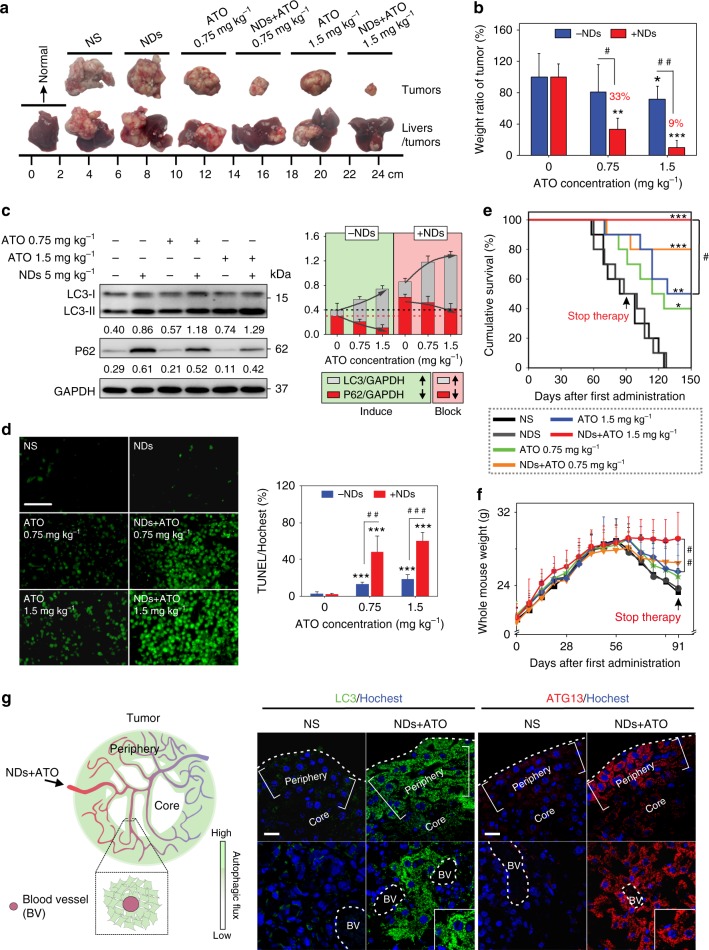
NDs API improves the ATO-based therapy in vivo. HepG2 tumor-bearing nude mice were administered intravenously (i.v.) with NS (200 μL), NDs (5 mg kg^−1^), ATO (0.75 mg kg^−1^), ATO (1.5 mg kg^−1^), NDs–0.75 mg kg^−1^ ATO mixture, or NDs–1.5 mg kg^−1^ ATO mixture daily for 5 consecutive days every week, with 13-week cycles. **a** Images of livers/tumors or excised tumors from treated tumor-bearing mice. **b** Weight ratio of tumors from treated mice (*n* = 10 for NS, 0.75 mg kg^−1^ ATO and 1.5 mg kg^−1^ ATO-treated groups, *n* = 8 for NDs–0.75 mg kg^−1^ ATO or NDs–1.5 mg kg^−1^ ATO-treated groups, *n* = 7 for NDs-treated group). **P < 0.01, ***P < 0.001 by *t*-test, significantly different from NS; ^#^*P* < 0.05, ^##^*P* < 0.01 by *t*-test, significantly different from ATO. **c** Immunoblots of autophagy-related proteins LC3-II, p62 (left); semi-quantified analysis (*n* = 3) in tumor tissues from mice after treatment (right). GAPDH was used as the loading control. Normalized band densities were shown below each band. **d** Representative images of TUNEL stains and its quantification by Image J. Scale bar: 50 μm. ***P < 0.001 by *t*-test, significantly different from NS; ^##^*P* < 0.01, ^###^*P* < 0.001 by *t*-test, significantly different from ATO. **e**, **f** Survival curves (**e**, *n* = 10) and whole body weight curves (**f**) of nude mice after various treatments (*n* = 20). *P < 0.05; **P < 0.01, ***P < 0.001 by *t*-test or one-way analysis of variance (ANOVA) using SPSS, significantly different from NS; ^#^*P* < 0.05, ^##^*P* < 0.01 by *t*-test or one-way analysis of variance (ANOVA) using SPSS, significantly different from ATO. **g** Immunostaining of LC3 and ATG13 in tumor tissues of NDs–1.5 mg kg^−1^ ATO-treated mice. Schematic showing (left) and imaging (right) indicate that LC3/Atg13 puncta are in the periphery and around the blood vessels inside the tumors. Scale bars: 20 μm. Error bars are s.d.

**Fig. 5:**
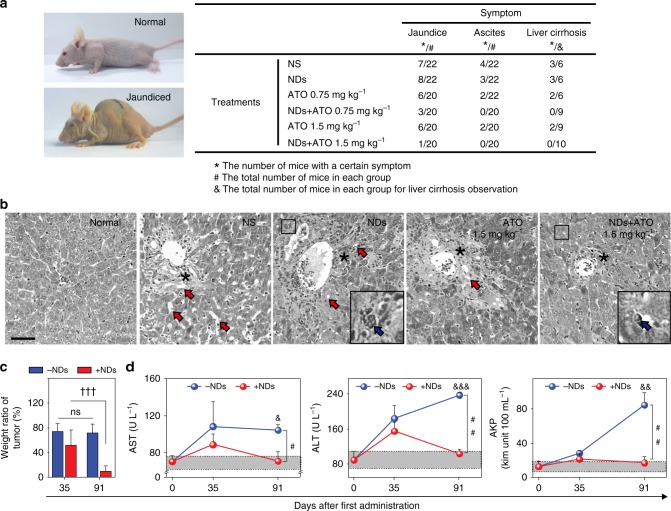
Therapeutic analysis of NDs–ATO combination therapy in short and long terms. HepG2 tumor-bearing nude mice were administered intravenously (i.v.) with ATO (1.5 mg kg^−1^) or NDs–ATO mixture daily for 5 consecutive days every week, with 5- or 13-week cycles (*n* = 8 and *n* = 20 for short- and long-term groups, respectively). **a** The left panel is photographs of normal and jaundiced tumor-bearing mice. The right panel is the table of a certain symptom number in tumor-bearing mice after long-term treatment. **b** Histopathology evaluation of liver tissues in orthotopic liver cancer-bearing nude mice after long-term ATO or NDs–ATO mixture treatment. Inflammatory cells infiltration around the central vein is indicated with an asterisk. Cellular vacuolization is indicated with red arrows. Cells containing NDs are indicated with blue arrows. Scale bar: 100 μm. **c** Weight ratio of tumors from treated mice. ^†††^*P* < 0.001 by *t*-test, significantly different from 35 days. ns not significant. **d** Analysis of biochemical parameters in serum. The region rendered in grey represents the range observed in normal nude mice. ^#^*P* < 0.05, ^##^*P* < 0.01 by *t*-test, significantly different from ATO; ^&^*P* < 0.05, ^&&^*P* < 0.01, ^&&&^*P* < 0.001 by *t*-test, significantly different from normal. Error bars are s.d.

Having demonstrated the excellent biocompatibility and tumor-targeting ability of this NDs/ATO co-treatment therapy, we explored whether NDs autophagy inhibitor could improve ATO-based therapy in vivo. Normal saline (NS), NDs, ATO, or NDs–ATO mixture were injected intravenously daily for 5 consecutive days every week to situ tumor-bearing nude mice established above (Supplementary Fig. 14). At 91 days after the first administration, compared to NS group, the NDs/ATO combination reduced the tumor weight to ~33%, while treatments with NDs or ATO (0.75 mg kg^−1^) alone did not significantly affect the tumor weight. For the 1.5 mg kg^−1^ ATO group, the addition of NDs reduced the tumor weight from 67% to 9%, suggesting a synergistic tumoricidal effect (Fig. 4a, b and Supplementary Fig. 15). Also importantly, co-administration of NDs/ATO led to greatly reduced advanced malignant liver tumor-associated symptoms, including cirrhosis, jaundice, and ascites (Fig. 5a, Supplementary Fig. 16 and Supplementary Table 3). Histological observation in multiple tissues showed that compared to other treatment regimes, co-treatment of NDs and 1.5 mg kg^−1^ ATO induced the least inflammatory cells infiltration around the central vein of liver tissues, no cellular vacuolization in liver tissue or glomerulus, and also no central necrosis of spleen tissue (Supplementary Fig. 17). Combined with biochemical parameter and major organ coefficient analysis (Supplementary Fig. 18 and Supplementary Table 4), we conclude that NDs/ATO co-treatment led to mitigation of malignant liver tumor-associated liver, kidney, and spleen failure. All these data further revealed the improved therapeutic efficacy of the NDs/ATO treatment.

Next, we explored the survival rate and health state of treated mice. In the group with the co-treatment of NDs and 1.5 mg kg^−1^ ATO, mice survived over 150 days without a single death; while all mice in negative controls (NS or NDs treatment groups) died within 125 days. Of note, treatment with ATO alone at various doses offered limited survival benefit compared to negative controls (Fig. 4e). More significantly, we found approximately 80% of mice in NDs–1.5 mg kg^−1^ ATO co-treatment group survived for over 20 weeks after stopping therapy. As a comparison, all mice in either the negative control or ATO-treated groups had succumbed to disease, and no more than 30% achieving long-term survival. Additionally, histological analysis, body weight curves, and coefficients of major organs suggested that the co-treatment regimen had no obvious side effects (Figs. 4f, 5b, Supplementary Figs. 17, 19 and Supplementary Table 4).

### NDs–ATO combination therapy in short and long terms

To further compare the therapeutic potential of NDs–ATO mixture with free ATO in relatively short and long terms, tumor weight ratio analysis and liver damage-associated serum biochemical test were carried out at 35 and 91 days after the first administration. We found that the weight ratio of tumor in NDs–ATO co-treatment group dramatically decreased with time, while that in ATO treatment group exhibited no obvious change with time (Fig. 5c), which confirmed the role of NDs in conferring resistance to ATO monotherapy. More interestingly, all biochemical parameters in NDs–ATO co-treatment group, which increased more or less at 35 days, eventually recovered to the range observed in normal mice at 91 days. In contrast, these parameters in the ATO-treatment group significantly increased along with the time (Fig. 5d). In addition, NDs–ATO have higher therapeutic efficacy than CQ–ATO. Tumor weight ratio analysis was carried out at 91 days after the first administration. We found that NDs–ATO reduced the tumor weight by ~91%, whereas it was only of ~58% for CQ–ATO (Fig. 4b and Supplementary Fig. 20). The lower efficacy of CQ is possibly due to the fact that the acidic pH in the tumor tissue hampers the CQ effects on autophagy^22^. All these data collectively suggested the high potential of using this NDs–ATO combination regime for further clinical applications.

### Molecular mechanism for NDs API-enhanced ATO therapy of liver tumors

**Fig. 6:**
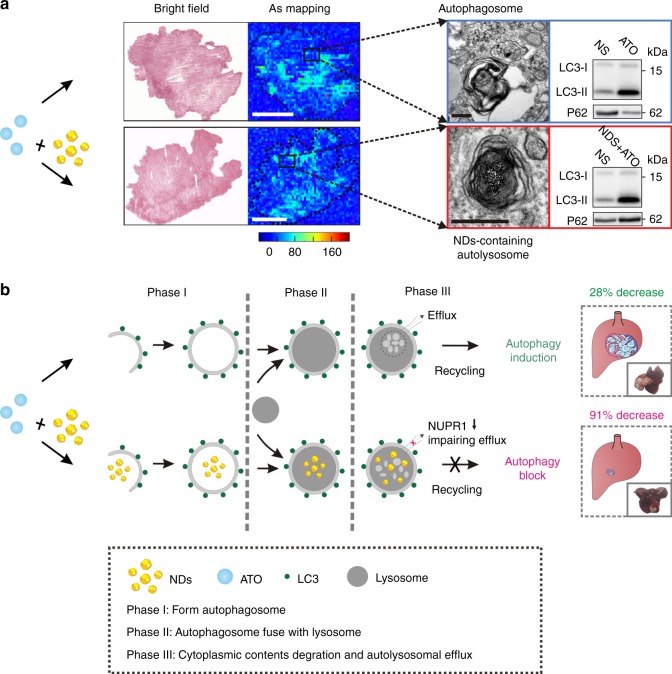
Molecular mechanism for NDs API-enhanced ATO therapy of liver tumors. **a**, **b** Experimental (**a**) and schematic showing (**b**) of combination therapy with ATO and NDs for solid tumor treatment by modulating cell autophagy event. Left panel in **a**, Arsenic distribution in tumor tissues by XRF. Scale bars: 2 mm. Right panel in **a**, TEM images of tumor sections (left) and autophagy-related protein expression in tumor tissues (right). Scale bars: 300 nm

We reason that the safety and potent efficacy of NDs–ATO arise from the organ-specific autophagic inhibition of NDs. We performed immunostaining of LC3II and ATG13 in tumor-bearing mice, two biomarkers of the autophagic flux in tumors^20,35^. We found that treated mice showed obvious LC3II and ATG13 puncta, especially in the periphery and around the blood vessels inside the tumors (Fig. 4g), which have a rich vascular network^36,37^. Hence, we confirmed the regulation of autophagic flux in tumors after the NDs–ATO co-treatment. Both western blot analysis and TEM images further confirmed that NDs effectively inhibited ATO-induced autophagy in hepatocellular carcinomas tissues (Figs. 4c; 6a, right). TEM analysis of tumor tissues also revealed the accumulation of NDs at the tumor site, possibly due to the enhanced permeability and retention (EPR) effect (Fig. 6a, right). In addition to tumor, immunofluorescence observation in liver and spleen tissues revealed that NDs–ATO co-treatment also regulated the autophagic flux in these tissues (Supplementary Fig. 21). Western blot analysis of liver and spleen tissues further confirmed this result (Supplementary Fig. 22). TUNEL staining in tissue sections showed that apoptosis increased as a result of in-situ autophagy inhibition (Figs. 4c, d; 6a, right). We further demonstrated that the increased efficacy was not due to the direct interactions between ATO and NDs. By employing synchrotron-based micro X-ray fluorescence (μXRF) microscopy and inductively coupled plasma optical emission spectrometry (ICP-OES), we examined the level of the As element in HepG2 (Supplementary Figs. 23 and 24), and found that the use of NDs in the ATO-treated HepG2 did not significantly affect the intracellular amount of As, which suggested that NDs and ATO were physically separate. Similarly, XRF mapping of tumor tissues did not show apparent increase of the ATO concentration (Fig. 6a, left and Supplementary Fig. 25). We note that the lack of specific NDs–ATO interaction is beneficial since the maintenance of low-concentration ATO is critical for its safe administration and therapy^38^. Indeed, in-vitro high-dose (>8 μM) ATO–NDs co-treatment did not show significantly increased cell death, probably because cell apoptosis dominated at high-dose ATO (Supplementary Fig. 26); whereas in-vivo high-dose (3 and 4 mg kg^−1^) ATO treatment resulted in significant weight loss of mice as well as limited therapeutic benefit (Supplementary Figs. 19 and 27).

## Discussion

We have established that NDs are a type of NAPI with high potency and biocompatibility. NDs have shown remarkable ability to allosterically enhance the tumoricidal effect of ATO in liver cancer models. We find that the low efficacy of ATO treatment (~28% carcinoma decrease) is greatly improved to ~91%, which arises from the high-level sensitization of ATO using NDs co-administration (Fig. 5c). Combination therapy-treated mice show 100% survival rate in 150 days with noticeably reduced advanced liver carcinoma-associated symptoms. More importantly, ~80% of post-therapy mice survive for over 20 weeks. In addition to their autophagic inhibition ability, NDs have several intrinsic properties that are also beneficial for their therapeutic applications. First, NDs were more safe and potent than existing chemical inhibitors (e.g., CQ). Second, NDs were autophagy inhibitors with excellent specificity and selectivity. We demonstrate that NDs function as autophagy inhibitors through impairing NUPR1-mediated autolysosomal efflux in cells (Fig. 6b). In particular, NDs have unique tissue-specific effects on autophagy (mainly tumor, liver, and spleen), thereby providing a promising approach for cancer therapy. Third, the lack of specific interactions between NDs and ATO avoids As accumulation in vivo and prevents adverse effects of ATO. Given that the interplay between autophagy and apoptosis is ubiquitous in tumors, the use of NDs, and probably more generally NAPI, provides a new solution not only to ATO-based therapy in solid tumors but to develop new treatment regimens for circumventing resistance in cancer therapy.

## Methods

### Materials

NDs with individual sizes of 2–10 nm, which are synthesized by detonation techniques, were supplied by Gansu Gold Stone Nano. Material. Co. Ltd. (Gansu, China). The unique crystal profile of the NDs was confirmed using an automatic X-ray diffractometer equipped with CuKа (1.541 Å) radiation (40 kV, 40 mA). TEM images showed that the size of the majority of ND clusters was about 40–200 nm (Supplementary Fig. 1a)^39^. GO with a mean thickness of 1.1 nm and lateral dimension ranging from 50 to 300 nm was prepared by the same method used in our previous work^40^. Briefly, large GO was prepared from purified natural graphite by using a modified Hummer’s method^41^. To obtain the uniform size distributed small GO nanosheets (called GO in the text), the as-prepared large GO should be further oxidized. After oxidation, GO was resuspended and dialyzed in Millipore water for 3 days, and then sonicated for 2–3 h to obtain stable, well-dispersed GO stock solution. The details for characterization are shown in Supplementary Fig. 1c. 10 nm Fe_2_O_3_ NPs were purchased from Ocean NanoTech. 20 nm Gold nanoparticles (AuNPs), 20 nm Fe_3_O_4_ NPs, and 10 nm CdSSe/ZnS core/shell QDs were purchased from Hangzhou Najing Technology Co. Ltd. (Supplementary Fig. 1a, b).

ATO was obtained from Sigma-Aldrich, USA. Stock solution of ATO was prepared with 0.1 M NaOH and was used to make serial dilutions. All other chemicals used were of analytical grade.

### Cell culture and treatment

HepG2 liver carcinoma cells (ATCC) were grown in RPMI1640 (Gibco) cell culture medium supplemented with 10% fetal bovine serum (FBS), and the resultant cell suspension (7 × 10^4^ cells mL^−1^) was dispensed into 24-well plates and incubated overnight to allow for cell adherence. After washing twice with phosphate buffered saline (PBS), cells were exposed to ATO, NDs, AuNPs, Fe_3_O_4_ NPs, CQ, NDs–ATO mixture, AuNPs–ATO mixture, Fe_3_O_4_–ATO mixture, or CQ–ATO mixture for 48 h as required.

NB4 leukemia cells (ATCC) were grown in RPMI1640 (Gibco) cell culture medium supplemented with 10% FBS, and the resultant cell suspension (1 × 10^5^ cells mL^−1^) was dispensed into 24-well plates and incubated overnight to allow for cell adherence. After washing twice with PBS, cells were exposed to ATO, NDs, CQ, NDs–ATO mixture, or NDs–ATO mixture for 48 h as required.

### Tumor models and treatment

Nude mice (male, 18–22 g) were purchased from Shanghai SLAC Laboratory Animal Co. Ltd., China and kept in pathogen-free conditions (18–22 °C, 50–70% relative humidity, 12 h light–dark cycle). All animal experiments were conducted in accordance with the Institute’s Guide for the Care and Use of Laboratory Animals and were approved by the ethical committee of Shanghai University of Traditional Chinese Medicine (Approval No. ACSHU-2014-200, approved on 16 July, 2014).

For the orthotopic liver cancer transplantation model establishment, 2 × 10^6^ HepG2 cells in 200 μL NS were subcutaneously injected in the right forelimbs of nude mice^42^ to form the tumor-supplying mice. The experimental nude mice were anesthetized and a midline incision was made to expose the liver. The orthotopic tumors were formed by implanting the tumor bits under the envelope of the mice liver, and then the liver and abdomen was closed with silk sutures^43^ tail vein injected with 200 μL NS, 5 mg kg^−1^ NDs, 0.75 mg kg^−1^ ATO, 1.5 mg kg^−1^ ATO, NDs–0.75 mg kg^−1^ ATO mixture, NDs–1.5 mg kg^−1^ ATO mixture, 10 mg kg^−1^ CQ, CQ–1.5 mg kg^−1^ ATO, 3 mg kg^−1^ ATO, or 4 mg kg^−1^ ATO for 5 consecutive days every week, with 5- or 13-week cycles.

### Western blotting analysis

Cells were cultured in 24-well plates to approximately 80% of confluence and treated with various materials as required. After washing twice with PBS, cells were harvested by SDS-loading sample buffer. Protein samples were analyzed by 10% or 12% SDS-PAGE (as appropriate) and blotted to PVDF membranes. The blots were blocked for 30 min using 6% nonfat milk in PBST (PBS containing 0.1% Tween 20) buffer and then incubated overnight at 4 °C with the primary antibodies as required: anti-LC3 (1:1000 dilution, Novus, NB100-2220), p62 (1:1000 dilution, Abcam, ab91562), ATG5 (1:1000 dilution, Cell Signaling, 12994S), ATG7 (1:1000 dilution, Cell Signaling, 8558S), NUPR1 (1:1000 dilution, Abcam, ab6028), SNAP25 (1:1000 dilution, Abcam, ab109105), VAMP8 (1:1000 dilution, Santa Cruz, SC166820), cathepsin D (1:1000 dilution, Santa Cruz), cathepsin B (1:1000 dilution, Santa Cruz, SC-377299), or glyceraldehyde 3-phosphate dehydrogenase (GAPDH) (1:1000 dilution, Cell Signaling, 2118S). After extensive washing, the blots were probed with a goat anti-rabbit/mouse horseradish peroxidase-conjugated antibody (1:1000 dilution, CST, 7074S/7076S) for 1 h. The blots were then developed by incubation with chemiluminescence (ECL) plus and exposed to X-ray film. The densities of all bands were quantified with a computer densitometer (AlphaImagerTM 2200 System Alpha Innotech Corporation, San Leandro, GBBOX-chemi-XL1.4). The expression of GAPDH was used as the protein loading control.

Orthotopic liver tumors excised from mice after treatment were minced, and homogenized in protein lysate buffer. Debris was removed by centrifugation, and the levels of LC3 and p62 in lysates were measured as above.

Original uncropped scans of western blots included in main figures are shown in Supplementary Fig. 28.

### Immunostaining

For in vitro experiments, after treatment, HepG2 cells were fixed using 4% paraformaldehyde in PBS for 20 min at room temperature, washed twice in PBS, and blocked for 45 min at room temperature in PBS containing 6% BSA and 0.25% Triton X-100. Cells were then stained with rabbit anti-LC3 antibody (1:200 dilution, Novus) and FITC-labeled secondary antibody (1:2000 dilution, KPL).

For in vivo experiments, paraffin-embedded tissue sections were treated with improved citrate antigen retrieval solution (Beyotime) for 30 min at 100 °C. Following twice wash with PBS, they were treated with 0.25% Triton X-100 for 15 min and blocked with PBS containing 6% BSA for 45 min at room temperature. Then, tissue sections were stained with rabbit anti-LC3 antibody (1:200 dilution, Novus) or rabbit Atg13 antibody (1:200 dilution, Cell Signaling, 13468S) and Anti-rabbit IgG (H+L), F(ab’)2 Fragment (Alexa Fluor® 488 Conjugate) or Anti-rabbit IgG (H+L), F(ab’)2 Fragment (Alexa Fluor® 594 Conjugate) secondary antibody (1:300 dilution, Cell Signaling, 4412S/8889S).

All stained samples were visualized under a laser confocal microscope (Leica TCS SP5).

### Transmission electron microscopy

For in vitro experiments, after treatment, HepG2 cells were washed twice with PBS, and prefixed with a few of 2.5% glutaraldehyde in 0.1 M PBS. Then, the cells were collected using a cell scraper and centrifuged at 2000 rpm for 10 min. Cell aggregates were fixed in 2.5% glutaraldehyde for at least 2 h. Following a further wash with PBS, the cells were then dehydrated in a graded gradient ethanol series and embedded in Epon618. For in vivo experiments, orthotopic liver tumor bits excised from mice were immediately fixed in 2.5% glutaraldehyde in 0.1 M phosphate buffer (PB) and stored at 4 °C until embedding. Ultrathin sections of the embedded cells and tumor tissues were examined by transmission electron microscope (TEM, JEOL-1230; JEOL).

### Statistical analysis

All results are expressed as the mean ± standard deviation from triplicate experiments performed in a parallel manner. Statistical significance of the data was determined by *t*-tests or one-way analysis of variance (ANOVA) using SPSS. *P < 0.05; **P < 0.01; ***P < 0.001.

## Electronic supplementary material

Supplementary Information

## Data Availability

The data supporting the main findings of this study are available within the main article and its Supplementary Information (Supplementary Figs. 1–28 and Supplementary Tables 1–4) or from the authors upon request.
