# Supplementary material for: Nanodiamond autophagy inhibitor allosterically improves the arsenical-based therapy of solid tumors
Source: Nat Commun. 2018 Oct 19;9:4347. doi: 10.1038/s41467-018-06749-2 (PMC6195623; doi:10.1038/s41467-018-06749-2)
Supplement: Supplementary file 1 — Supplementary Information [file 41467_2018_6749_MOESM1_ESM.pdf]

Nanodiamond autophagy inhibitor allosterically improves the arsenical-based therapy of solid tumors

Zhifen Cui[1#], Yu Zhang[1#], Kai Xia[1#], Qinglong Yan[1#], Huating Kong[1], Jichao Zhang[1], Xiaolei Zuo[1,2], Jiye Shi[3], Lihua Wang[1]*, Ying Zhu[1]* and Chunhai Fan[1,4]*

[1]Division of Physical Biology and Bioimaging Center, Shanghai Synchrotron Radiation Facility, Shanghai Institute of Applied Physics, Chinese Academy of Sciences, Shanghai 201800, China.

[2]Renji Hospital, Shanghai 200001, China.

[3]Kellogg College, University of Oxford, Banbury Road, Oxford, OX2 6PN.

[4]School of Life Science and Technology, Shanghai Tech University, Shanghai 200031, China.

[#]These authors contributed equally to this work.

*To whom correspondence should be addressed: C.F. (Tel: +86 21 3919 4129, Fax: +86 21 3919 4022, E-mail: fchh@sinap.ac.cn), Y.Z. (Tel: +86 21 3919 4007, Fax: +86 21 3919 4022, E-mail: zhuying@sinap.ac.cn) or L.W. (Tel: +86 21 3919 4609, Fax: +86 21 3919 4022, E-mail: wanglihua@sinap.ac.cn).

Supplementary methods.

shRNA transfection

A pLKO system (Sigma-Aldrich, St. Louis, MO) was utilized for RNAi of autophagy related proteins. TRC numbers for shRNAs used are: ATG7 #1, ATG7 #2, ATG5 #1, ATG5 #2), non-target (Scramble) (Supplementary Table 1). Cells were transduced with lipo 3000 according to Lipofectamine^{TM} 3000 Transfection Reagent (Invitrogen). Level of targeted knockdown was determined by Western blot analysis (Supplementary Fig. 2).

Evaluation of dysfunction in autolysosomal processing

Dysfunction in autolysosomal processing, either an increase in autophagosome-lysosome flow or a decrease in autolysosomal clearance[1]. To investigate it, a tandem reporter construct mCherry–GFP–LC3 was used[2]. The green fluorescence of this tandem reporter is attenuated in the acidic pH lysosomal environment, whereas the mCherry is not. Thus this probe allows distinction between autolysosomes (GFP+/mCherry+ yellow puncta) induced by increased autophagosome-lysosome flow and autolysosomes (GFP−/mCherry+ red puncta) induced by decreased autolysosomal clearance. After 24 h transfection with mCherry–GFP–LC3 plasmid (Addgene), cells were treated with CQ or NDs for an extra 48 h.

Cytotoxicity analysis

Cell viability was determined by the MTT [3-(4,5-dimethylthiazol-2-yl)-2,5-diphenyltetrazolium bromide] (Sigma-Aldrich, Shanghai, China) assay and expressed as a percentage of ODtest/ODcontrol. All of the viability assessment data was based on three independent measurements.

Cells for fluorescence imaging were grown on 24-well plates with cover slips coated with Matrigel™ (BD Matrigel™ Matrix Basement Membrane, BD, Franklin Lakes, New Jersey, US). Then, the cells were washed twice with PBS and stained with calcein AM (Invitrogen, 2 μM, 20 min, λ_{ex} 488 nm, λ_{em} 515nm). Following a further wash with PBS, cells were imaged by confocal laser scanning microscopy (Leica TCS sp8 microscope).

Caspase-3 activity determination

The activity of caspase-3 was determined using the Caspase-3 activity kit (Beyotime, China) according to the manufacturer's protocol. Briefly, a reaction mixture of 10 μL cell lysate, 80 μL reaction buffer (20 mM Tris-HCl, pH 7.5, 1% NP-40), and 10 μL caspase-3 substrate (Ac-DEVD-pNA) (2 mM) were prepared and incubated at 37 °C for 4 h. Absorbance at 405 nm was measured using a microplate reader (Bio-Rad 680, USA).

Hematologic and biomedical marker analysis

Blood samples were collected in evacuated tubes containing EDTA, sodium citrate anticoagulants and non-anticoagulant agents. The blood analysis (biochemistry

and haematology) was carried out on SYSMEX XS-500i and Hitachi 7100, respectively.

NDs-XenoLight 770 preparation

NDs-770 was prepared following our previous protocals[1]. After conjugation with XenoLight 770 dyes, the zeta potential of NDs was shifted from 44.1 eV to 33.1 eV. In combination with the FTIR spectra (Supplementary Fig. 12), we confirmed that the NDs-CF770 conjugation has been well prepared. The fluorescent labeling rate calculated by indiGo software is about 0.21 nmol dye per mg NDs.

Blood circulation monitoring

Blood circulation was measured by drawing 10 μL blood from the nude mice post-injection of NDs-CF770-ATO mixture. The concentration of NDs in the blood was determined by the fluorescence spectrum of each blood sample using an In-vivo Xtreme Imaging system (BRUKER). The concentration of ATO in the blood was determined by inductively coupled plasma-mass spectrometry (ICP-MS, NexION 300D, PE-PerkinElmer) A series of dilutions of the NDs-CF770 in whole blood were measured to obtain a standard calibration curve (Supplementary Fig. 13). Blank blood sample without NDs-CF770-ATO treatment was measured to determine the blood auto-fluorescence level, which was subtracted from the fluorescence intensities of injected samples during the concentration calculation.

Biodistribution analysis

NDs covalently labeled with the near-infrared XenoLight 770 dye (PE-PerkinElmer) or equal amount of dye molecules were tail vein injected into mice, respectively. Whole-body imaging and detailed organ distribution studies (5 per group at each time point) were conducted by using the Maestro in vivo fluorescence imaging system (CRi Inc.).

TUNEL analysis

TUNEL staining was performed using the in situ death-detection POD kit (Roche Diagnostics) on paraffin-embedded orthotopic tumor sections per manufacturer's instructions. Hochest counterstain was used to quantify cells with intact nuclei. Results were analyzed by fluorescent microscopy (Zeiss Axioskop2 plus) and quantified with ImageJ (NIH).

Hepatotoxicity and nephrotoxicity studies

Serum levels of ALT, AST, ALP activities, Crea and BUN concentrations were determined according to the detection kit (Nanjing Jiancheng Bioengineering Institute, China). The results were expressed as U L^{-1}, U L^{-1}, kim unit 100 mL^{-1}, mg dL^{-1} and μg mL^{-1}, respectively.

Histopathological analysis

Paraffin-embedded liver, spleen, lung and kidney sections of orthotopic

tumor-bearing mice were stained with hematoxylin–eosin and examined by optical microscopy. The pathologist performing the visual analysis was blind to the grouping of mice.

Coefficient analysis of major organs

After weighing the body and tissues, the coefficients of liver, spleen, lung and kidney to body weight were calculated as the ratio of tissues (wet weight, mg) to body weight (g).

Synchrotron-based X-ray fluorescence microscopy

For *in vitro* experiments, the Mylar X-ray films (Hoffman, 12 μm thickness) were previously put into 24-well plates and sterilized by successive baths in 70% ethanol. HepG2 cell suspension was dispensed into 24-well plates and incubated overnight to allow for cell adherence to the thin films. After treatment, cells were fixed with a few of 4% paraformaldehyde in 0.1 M PBS. Following wash with PBS, the cells were then dehydrated in a graded gradient ethanol series and dried under air. The micro X-ray fluorescence (μXRF) microscopy was performed at the beamline BL15U1 of SSRF. Incident x-rays energy of 12 keV, obtained with a Si (111) monochromator, was chosen in order to excite the K-lines of X-ray fluorescence of elements from P to As. A light microscope was coupled to a computer for sample viewing and the sample platform was moved by a motorized X-ray mapping stage. A Kirkpatrick-Baez mirror system focused the x-ray beam to a spot size of 2.5 × 2.5 μm on the specimen, which

was raster-scanned. XRF from the specimen was captured with an energy dispersive silicon drift detector (Vortex, USA). From the analysis of the X-ray fluorescence spectrum for each pixel, a spatial image can be obtained for each element separately. Such an image represents a two-dimensional projection of the volumetric distribution of the elements. The vertical and horizontal pixel size was 2 μm each. Data collection time for each pixel was 2 s and fitting of the fluorescence data has been performed in batch processing using the PyMca 4.0.9 software.

For *in vivo* experiments, paraffin-embedded orthotopic tumor sections were placed on Mylar X-ray films. A Kirkpatrick-Baez mirror system focused the x-ray beam to a spot size of 100 × 100 μm on the specimen, which was raster-scanned. The vertical and horizontal pixel size was 100 μm each. Data collection time for each pixel was 4 s and fitting of the fluorescence data has been performed in batch processing using the same software. Other experimental conditions and parameter setting are the same as the *in vitro* experiments.

Inductively coupled plasma-mass spectrometry (ICP-MS)

For in vitro experiments, after treatment, cells were washed three times with PBS, trypsinized, collected and transferred to Enppendorf tubes. Aliquots of 100 μL were taken from the each sample to determine the number of cells, and the remainder of the cell suspensions was sonicated for 3-5 min. Following centrifugation at 12000 rpm for 15 min, the supernatant was diluted at appropriate dilution.

For in vivo experiments, blood and tissues including heart, liver, spleen, lung,

kidney and tumors were digested by HNO_3 and H_2O_2 mixture (v/v ratio is 7:1) at 120 °C until the mixed solutions became colorless and clear.

As concentration in all samples was analyzed by ICP-MS.

Supplementary figures and tables.

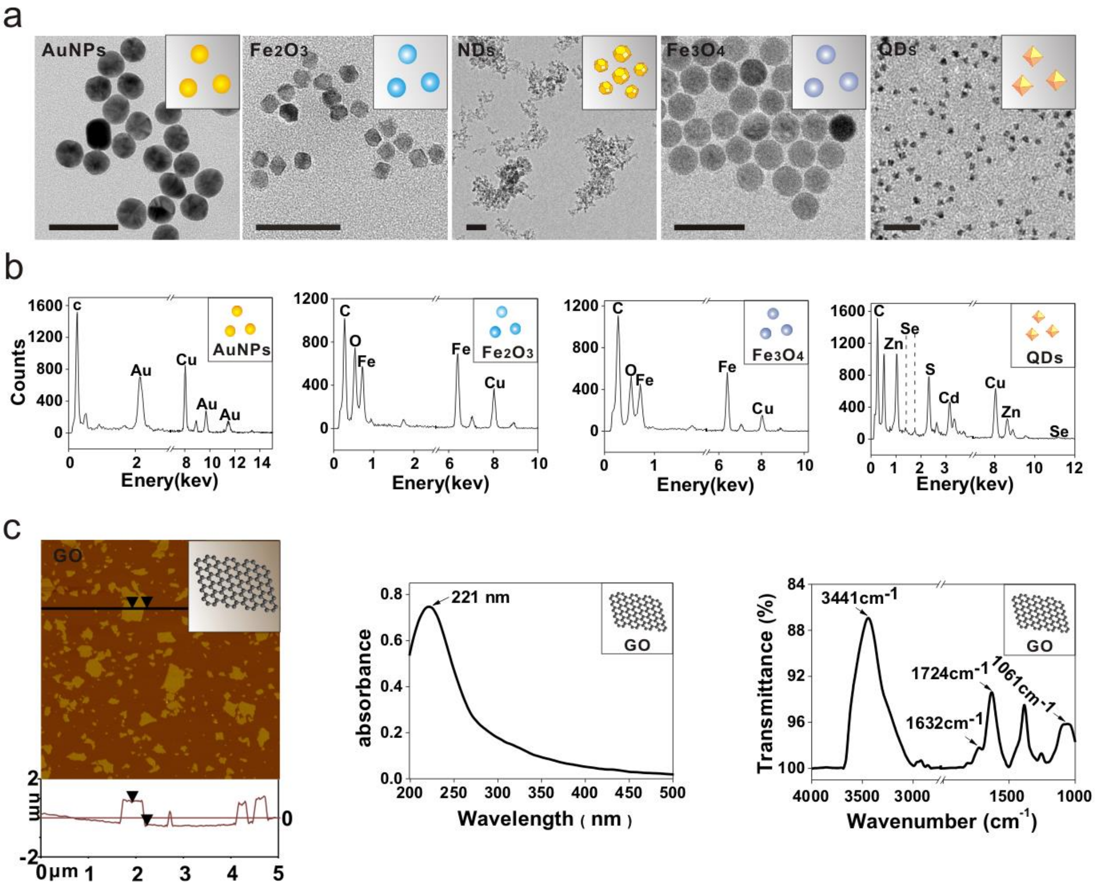

Supplementary Figure 1. Characterization of various nanoparticles (NPs). a, TEM images of various NPs. Scale bar: 50 nm. **b**, EDX analysis of TEM images. Sample was placed on a copper TEM grid for examination. **c**, AFM image (left), UV-Vis spectra (middle) and FTIR spectra (right) of graphene oxide (GO).

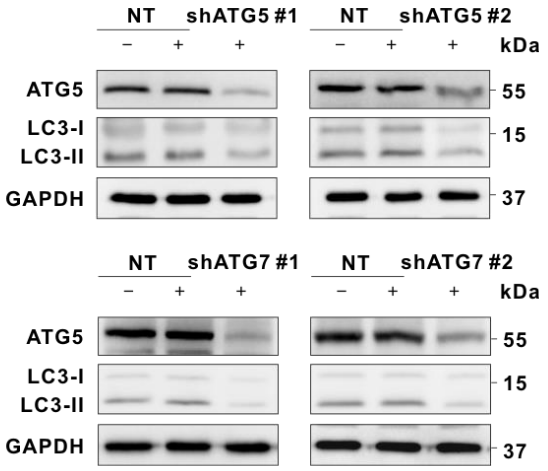

Supplementary Figure 2. Immunoblots show the effectiveness of ATG5 and ATG7 RNAi and resultant decrease of LC3II. NT: no-targeted RNAi. GAPDH was used as the loading control.

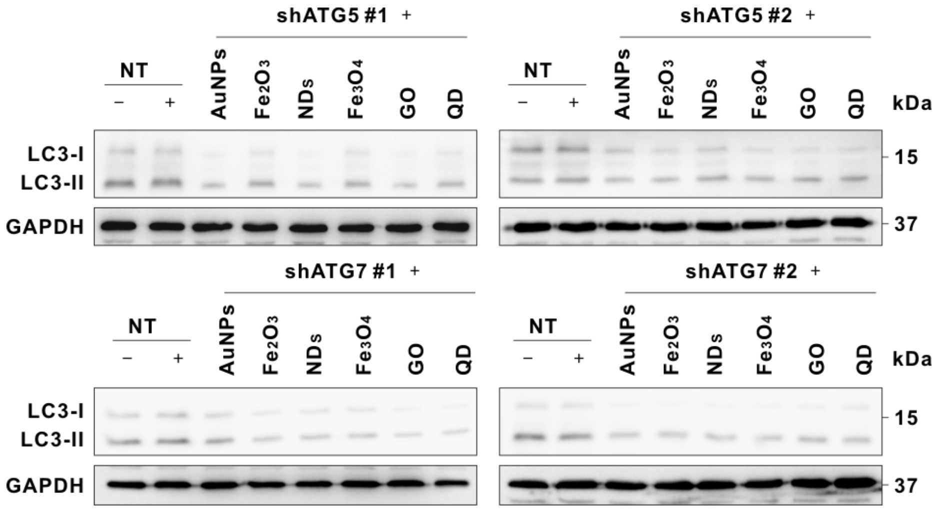

Supplementary Figure 3. Immunoblots for autophagy-related proteins LC3-II in cells after various nanoparticles treatment with ATG5 or ATG7 RNAi. GAPDH was used as the loading control.

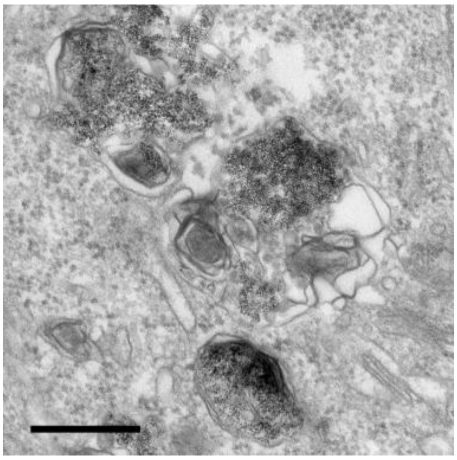

Supplementary Figure 4. **TEM image of NDs treated cells**. Scale bar: 400 μm.

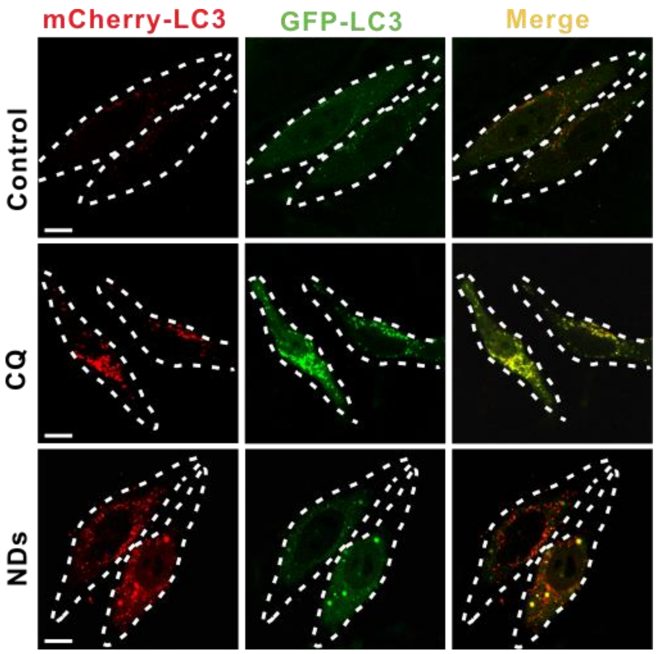

Supplementary Figure 5. Fluorescence images of mCherry-GFP-LC3 cells after incubation with CQ or NDs for 48 h (Autophagosomes: mCherry+/GFP+ yellow puncta; autolysosomes: mCherry+/GFP). Scale bars: 10 μm.

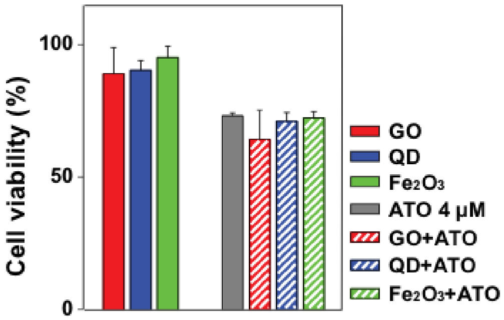

Supplementary Figure 6. The viability of HepG2 after incubation with ATO or various autophagy-induction NPs-ATO mixture for 48 h (n = 3; error bars are s.d.)**.**

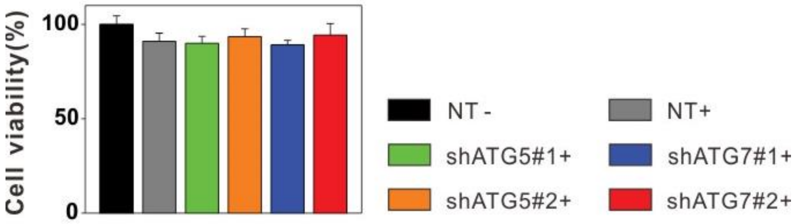

Supplementary Figure 7. Cell viability after 48 h NDs treatment with RNAi of autophagy proteins ATG5 and ATG7 (n = 3; error bars are s.d.). NT: no-targeted RNAi.

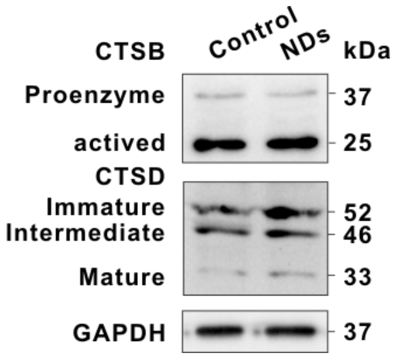

Supplementary Figure 8. Immunoblots for lysosomal proteases cathepsins B/D (CTSB and CTSD) in NDs treated cells. GAPDH was used as the loading control.

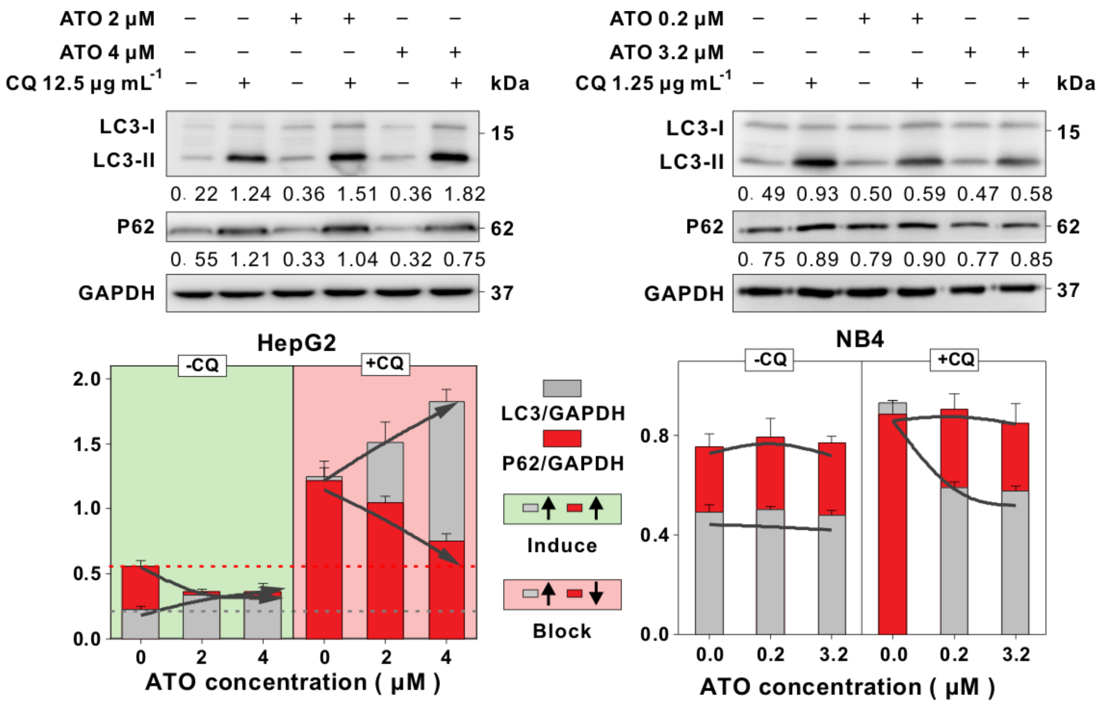

Supplementary Figure 9. Immunoblots of autophagy related proteins LC3-II, p62 (left); semi-quantified analysis (n = 3) in CQ, ATO or CQ-ATO mixture treated HepG2 and NB4 cells (right). GAPDH was used as the loading control. Normalized band densities were shown below each band. Error bars are s.d.

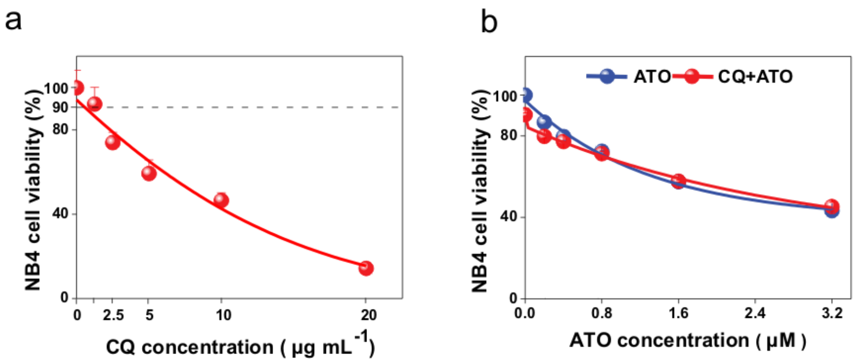

Supplementary Figure 10. Combined effects of ATO with clinical chemical inhibitor CQ in APL (NB4) cells. a, Cell viability after incubation with CQ at various concentration for 48 h (n = 3). The safe concentration of CQ for the cells is 1.25 μg mL^{-1}. **b**, Cell viability after incubation with ATO or CQ-ATO mixture for 48 h (n = 3). Error bars are s.d.

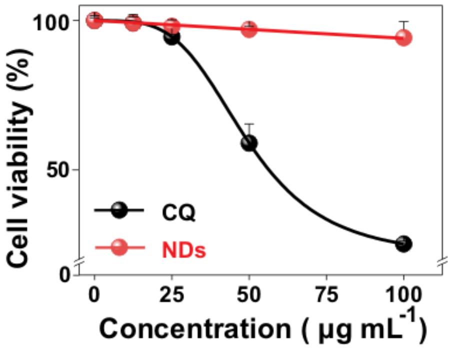

Supplementary Figure 11. Cell viability after incubation with NDs or CQ at various concentration for 48 h (n = 3; error bars are s.d.).

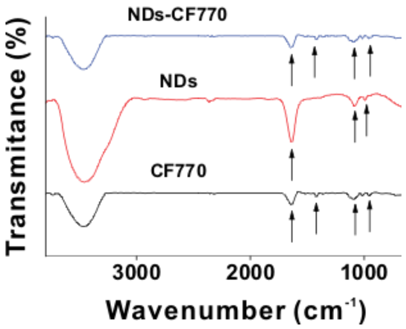

Supplementary Figure 12. FTIR spectra of CF770, NDs and NDs-CF770. Arrows show IR features of CF770 at 1641 cm^{-1}, 1421 cm^{-1}, 1085 cm^{-1} and NDs at 1131 cm^{-1}, 1258 cm^{-1}, 1325 cm^{-1}, respectively.

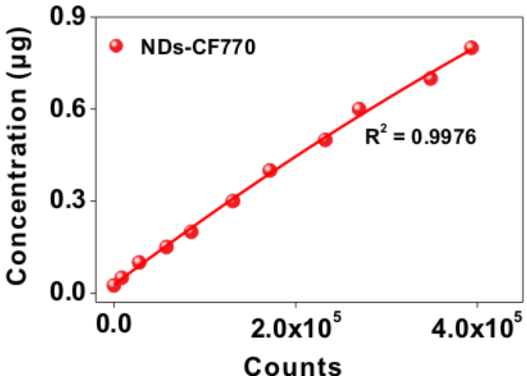

Supplementary Figure 13. Standard curves of NDs-CF770 in whole blood of mice.

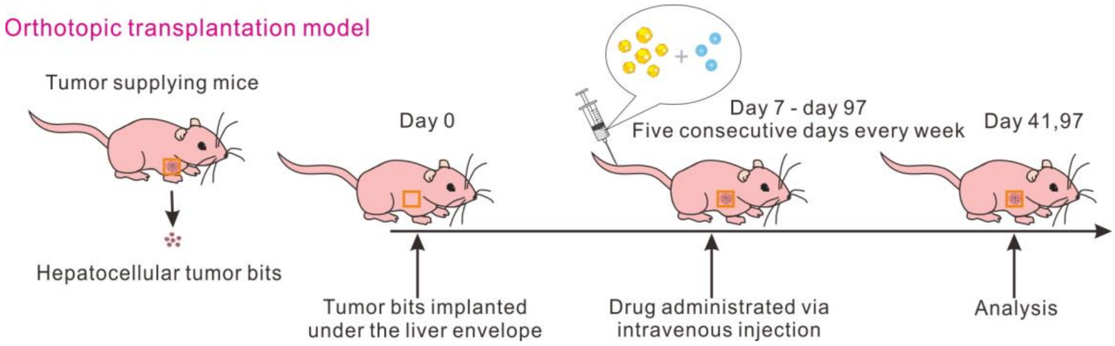

Supplementary Figure 14. General in vivo experimental design.

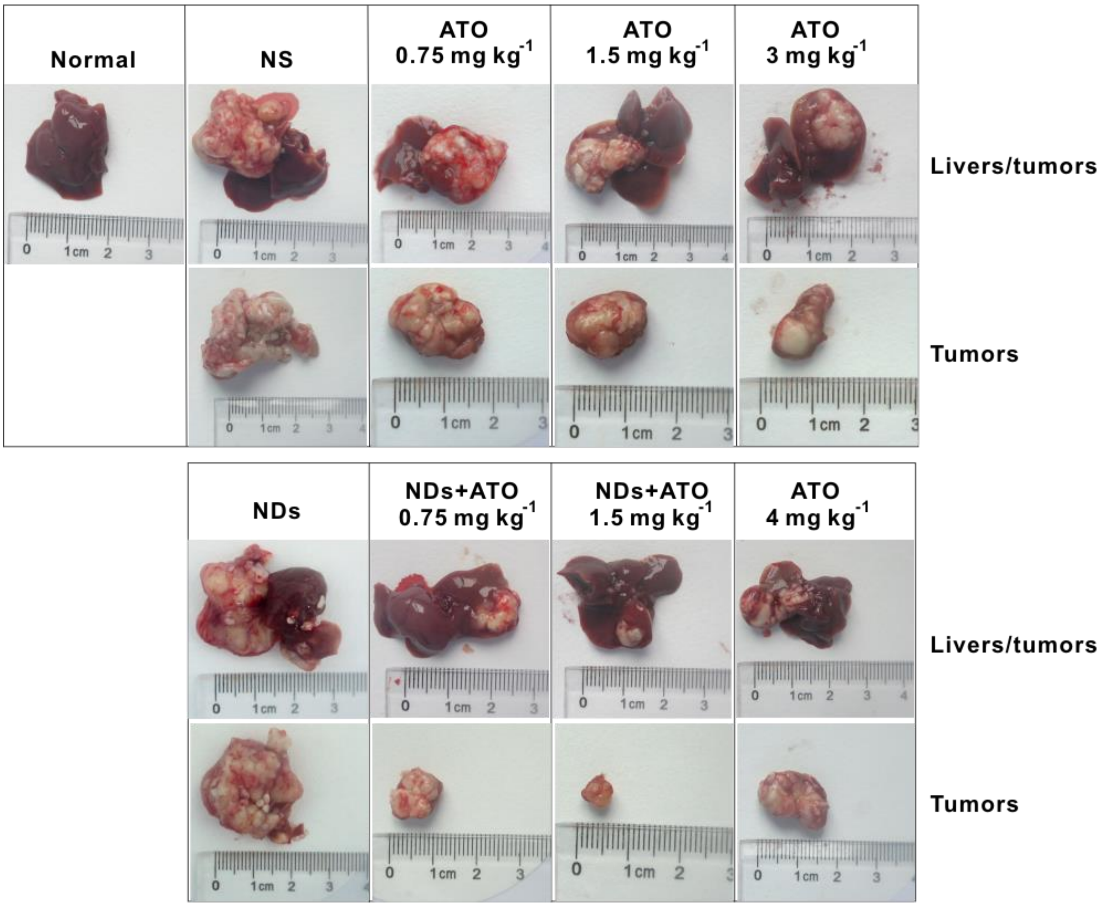

Supplementary Figure 15. Original photos of livers/tumors or excised tumors from nude mice after treatment. HepG2 tumor-bearing nude mice were administered intravenously (i.v.) with NS (200 μL), NDs (5 mg kg^{-1}), ATO (0.75 mg kg^{-1}), ATO (1.5 mg kg^{-1}), ATO (3 mg kg^{-1}), ATO (4 mg kg^{-1}), NDs-0.75 mg kg^{-1} ATO mixture or NDs-1.5 mg kg^{-1} ATO mixture daily for five consecutive days every week, with 13 week cycles.

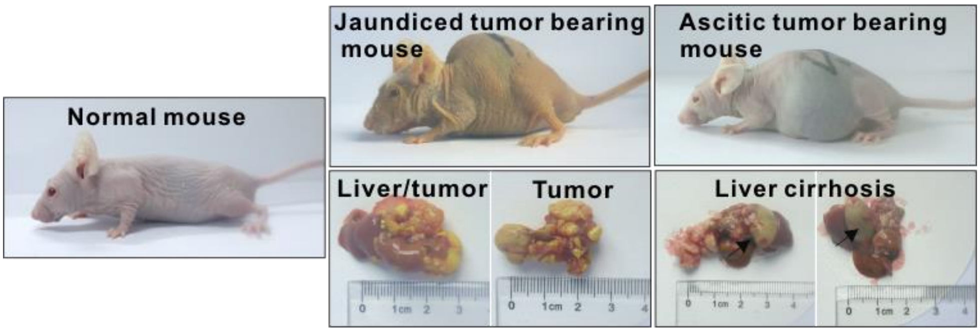

Supplementary Figure 16. Photos of mice, livers/tumors or excised tumors with certain advanced malignant liver tumor associated symptoms.

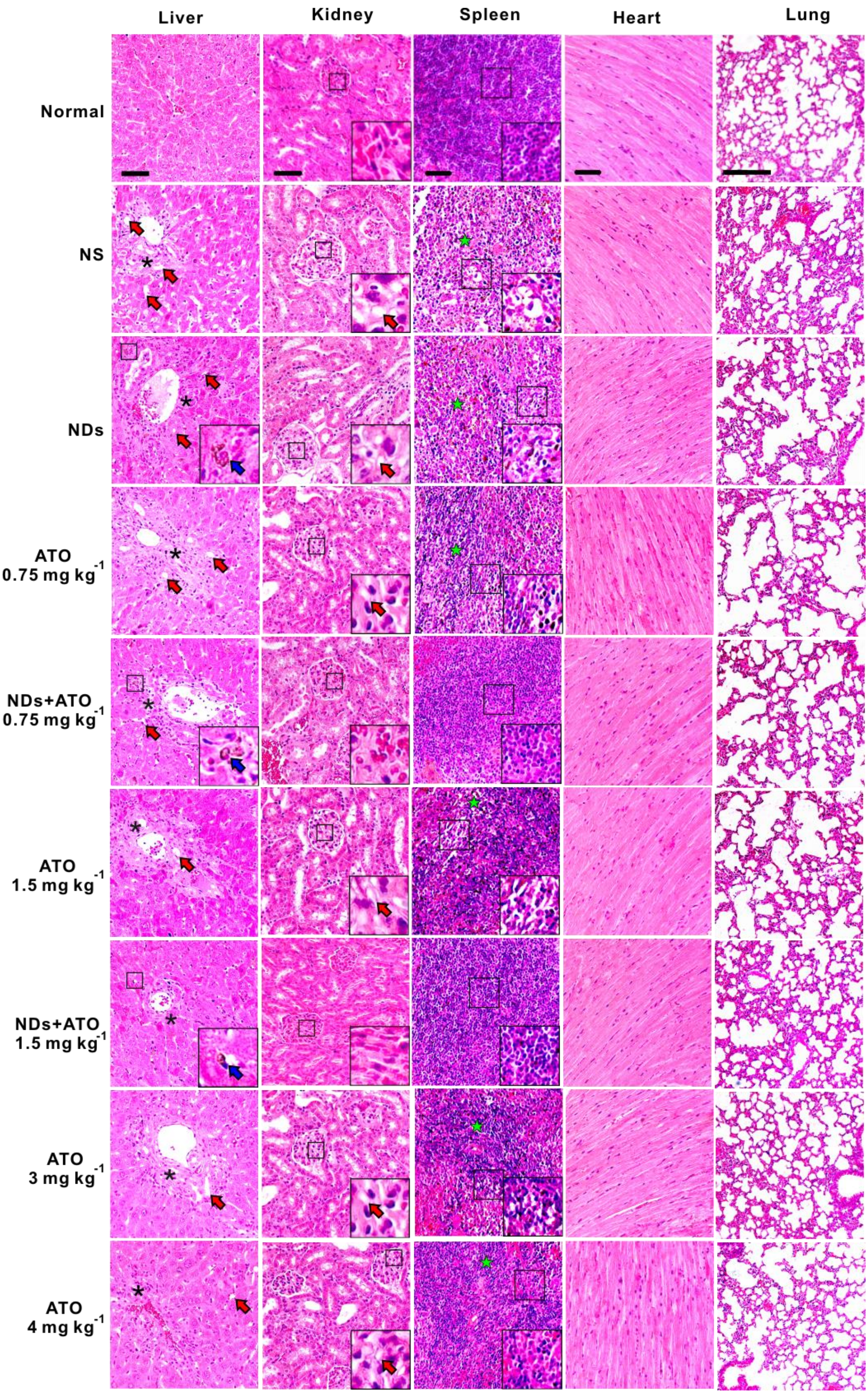

Supplementary Figure 17. Hematoxylin and eosin (H&E) histopathological

sections of liver, kidney, spleen, heart and lung tissues were analyzed after various treatments as indicated in Fig. S15. Inflammatory cells infiltration around the central vein of liver tissues are indicated with asterisk. Cellular vacuolization in liver tissue or glomerulus are indicated with red arrows. Cells containing NDs are indicated with blue arrows. Central necrosis of spleen tissue are indicated with green pentagram. Scale bars: 100 μm.

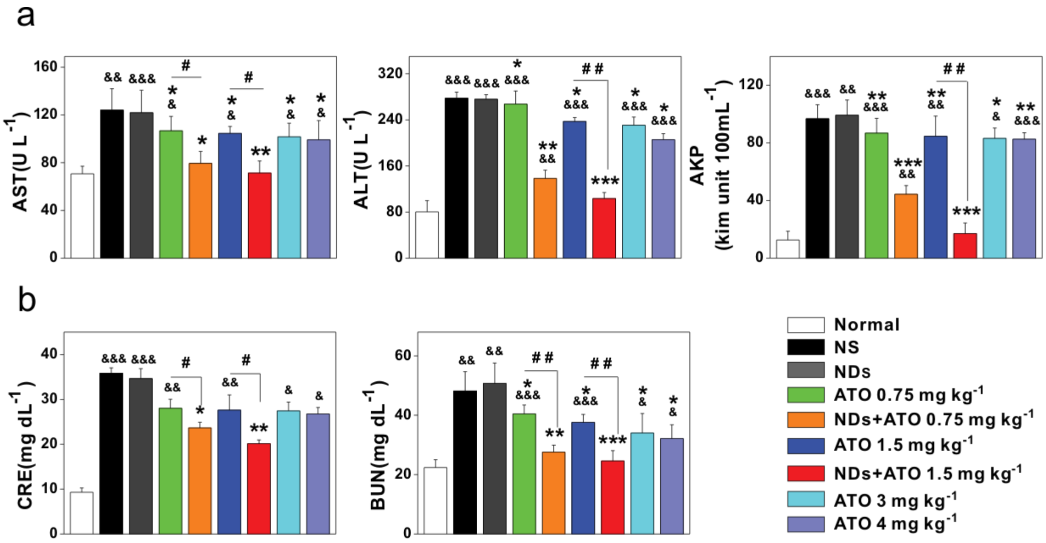

Supplementary Figure 18. Analysis of biochemical parameters in nude mice serum after various treatments as indicated in Fig. S15. a, Liver damage-associated serum biochemical parameters. **b**, Kidney damage-associated serum biochemical parameters. **P* < 0.05, ***P* < 0.01, ****P* < 0.001 by t-test, significantly different from NS. #*P*<0.05, ##*P*<0.01 by t-test, significantly different from ATO. &*P* < 0.05, &&*P* < 0.01 by t-test, significantly different from normal. Error bars are s.d.

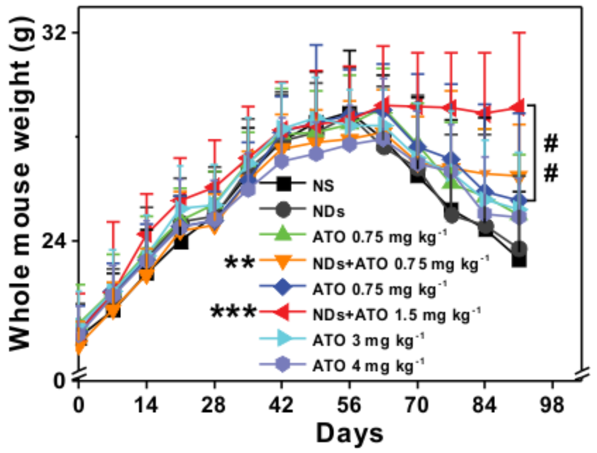

Supplementary Figure 19. Whole body weight curves of nude mice after various treatments as indicated in Fig. S15. *******P* < 0.01, ********P* < 0.001 by t-test, significantly different from NS. #*P*<0.05, ##*P*<0.01 by t-test, significantly different from ATO. Error bars are s.d.

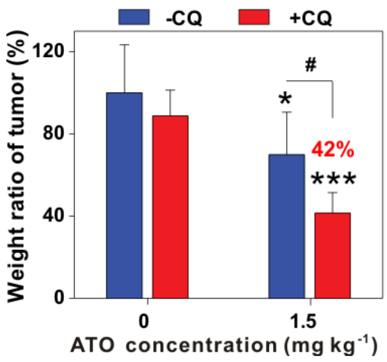

Supplementary Figure 20. HepG2 tumor-bearing nude mice were administered intravenously (i.v.) with NS (200 μL), CQ (10 mg kg^{-1}), ATO (1.5 mg kg^{-1}), CQ-1.5 mg kg^{-1} ATO mixture (n = 8 per group) daily for five consecutive days every week, with 13 week cycles. *P<0.05; ***P<0.001 by t-test, significantly different from NS; #P<0.05 by t-test, significantly different from ATO. Error bars are s.d.

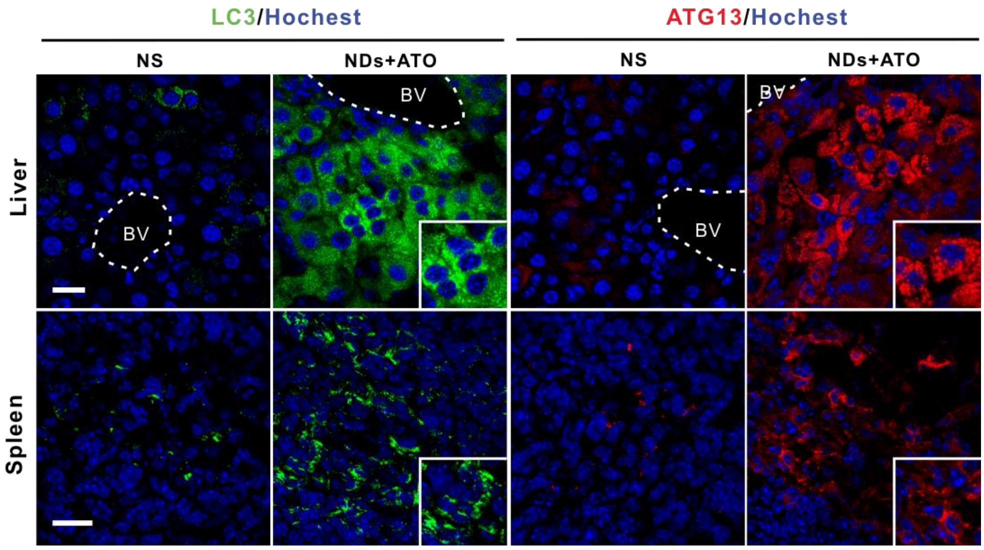

Supplementary Figure 21. Immunostaining of LC3 and ATG13 in liver and spleen tissues. HepG2 tumor-bearing nude mice were administered intravenously (i.v.) with NDs-1.5 mg kg^{-1} ATO mixture daily for five consecutive days every week, with 13 week cycles. Scale bars: 20 μm.

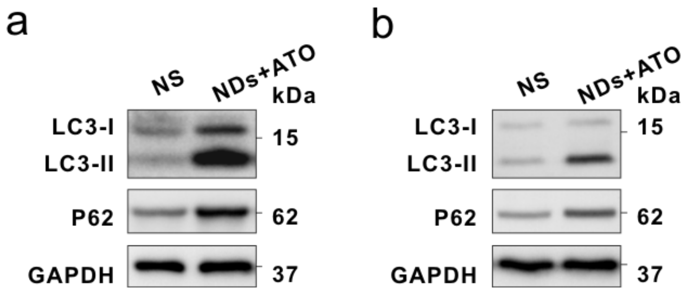

Supplementary Figure 22. Immunoblots of autophagy related proteins LC3-II and p62 in liver (a) and spleen (b) tissues from mice after NDs-1.5 mg kg^{-1} ATO treatment. GAPDH was used as the loading control.

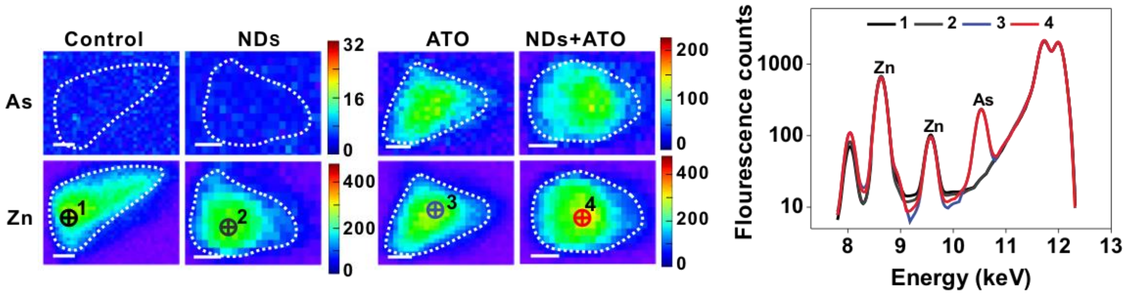

Supplementary Figure 23. Imaging of intracellular arsenic distribution by μXRF (Left). Elemental maps of arsenic (upper) and zinc (lower) are shown. The right panel is representative in situ X-ray emission spectra for arsenic and zinc of cells in left panel. Scale bars: 5 μm.

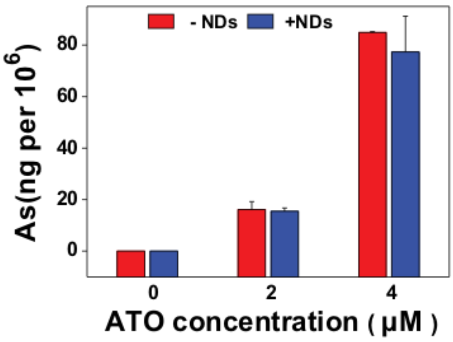

Supplementary Figure 24. Intracellular arsenic concentration in ATO or NDs-ATO mixture treated HepG2 cells determined by ICP-OES. Error bars are s.d.

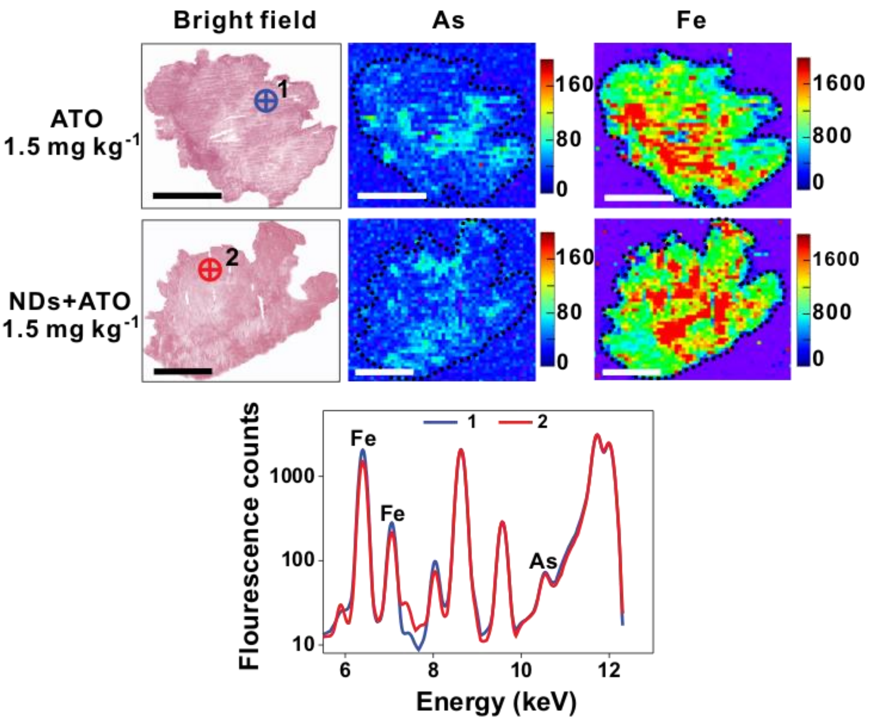

Supplementary Figure 25. Arsenic distribution in tumor tissues by XRF. HepG2 tumor-bearing nude mice were administered intravenously (i.v.) with ATO (1.5 mg kg^{-1}) or NDs-1.5 mg kg^{-1} ATO mixture daily for five consecutive days every week, with 13 week cycles. Upper: Elemental maps of arsenic (middle) and iron (right). Each X-ray fluorescence image is paired with its respective histological image (left). Lower panel is representative in situ X-ray emission spectra for arsenic and iron of tumor tissue in orthotopic liver cancer bearing nude mice in upper panel. Scale bars: 2 mm.

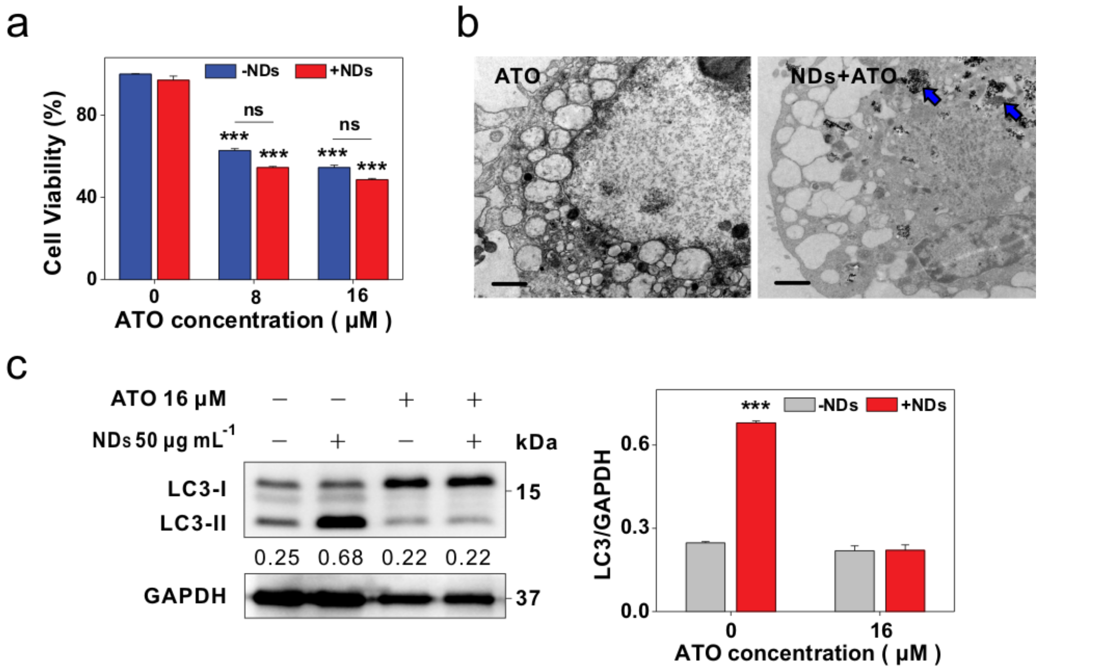

Supplementary Figure 26. NDs mediated autophagy inhibition in combination high-dose ATO treatment in HepG2 cells. a, Cell viability after incubation with ATO or NDs-ATO mixture for 48 h (n = 3). ***P < 0.001 by t-test, significantly different from control. ns, not significant. **b**, TEM images of ATO or NDs-ATO mixture treated HepG2 cells. NDs are indicated with blue arrows. The concentration of ATO is 16 μM. Scale bars: 1 μm. **c**, Immunoblots of autophagy related proteins LC3-II (left); semi-quantified analysis (n ＝ 3) in NDs, ATO or NDs-ATO mixture treated cells (right). GAPDH was used as the loading control. Normalized band densities were shown below each band. Error bars are s.d.

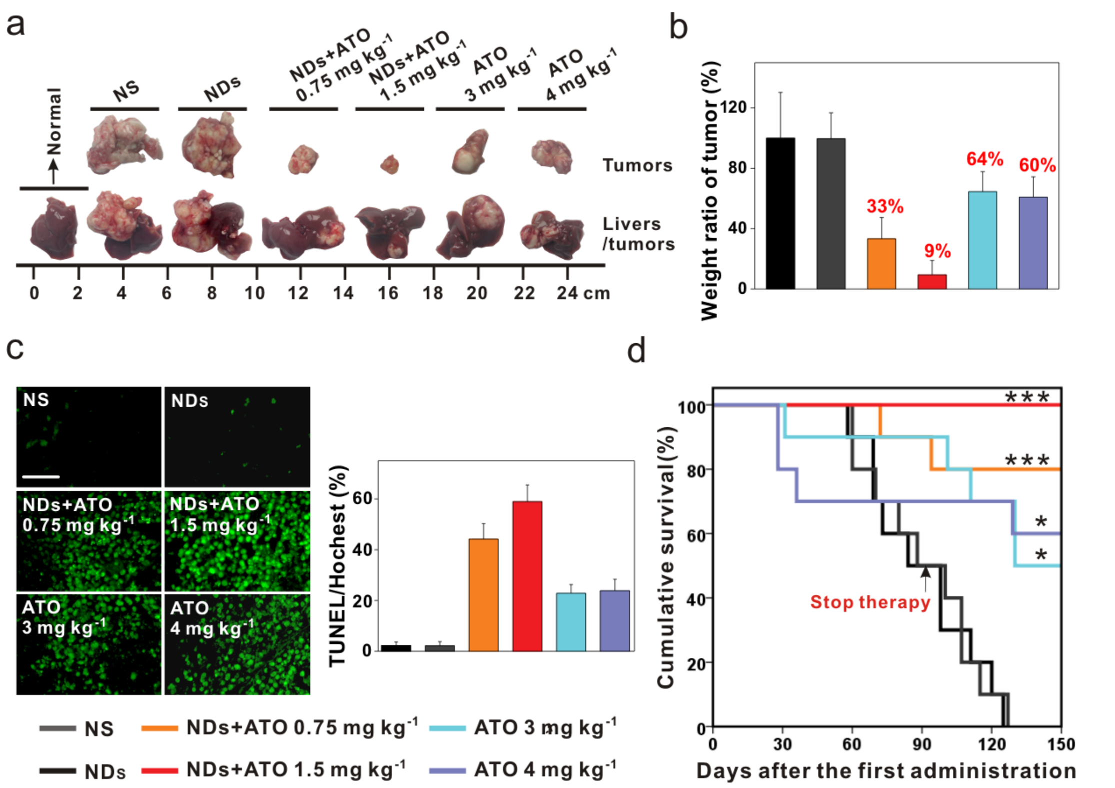

Supplementary Figure 27. NDs mediated autophagy inhibition in combination ATO treatment in orthotopic liver tumor transplantation models. HepG2 tumor-bearing nude mice were administered intravenously (i.v.) with NS (200 μL), NDs (5 mg kg^{-1}), ATO (3 mg kg^{-1}), ATO (4 mg kg^{-1}), NDs-0.75 mg kg^{-1} ATO mixture or NDs-1.5 mg kg^{-1} ATO mixture daily for five consecutive days every week, with 13 week cycles. **a**, Images of livers/tumors or excised tumors from treated mice. **b**, Weight ratio of tumors from treated mice (n = 10 for NS treated group, n = 8 for NDs-0.75 mg kg^{-1} ATO or NDs-1.5 mg kg^{-1} ATO treated groups, n = 7 for NDs or 4 mg kg^{-1} ATO treated group, n = 9 for 3 mg kg^{-1} ATO treated group). **c**, Representative images of TUNEL stains and its quantification by Image J. Data are represented as means ± SD. Scale bar: 50 μm. **d**, Survival curves of nude mice after treatments. **P* < 0.05;****P* < 0.001 by one-way analysis of variance (ANOVA) using SPSS, significantly different from NS. Error bars are s.d.

Fig. 1 a

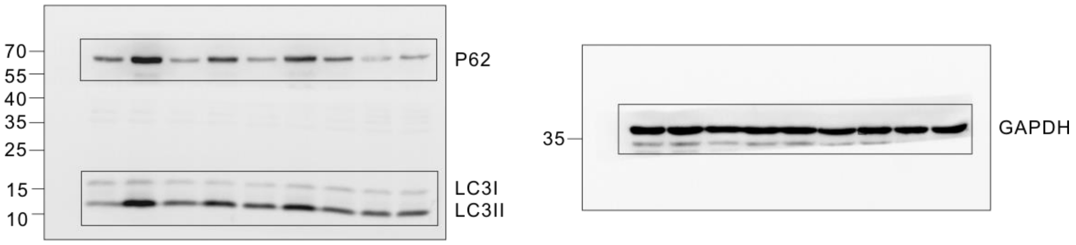

Fig. 1 c

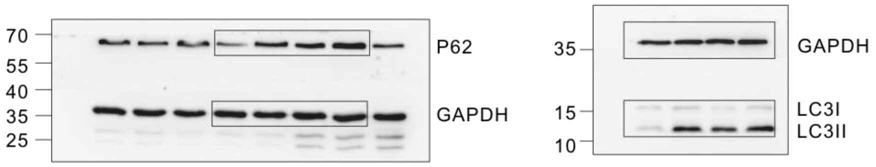

Fig. 1 e

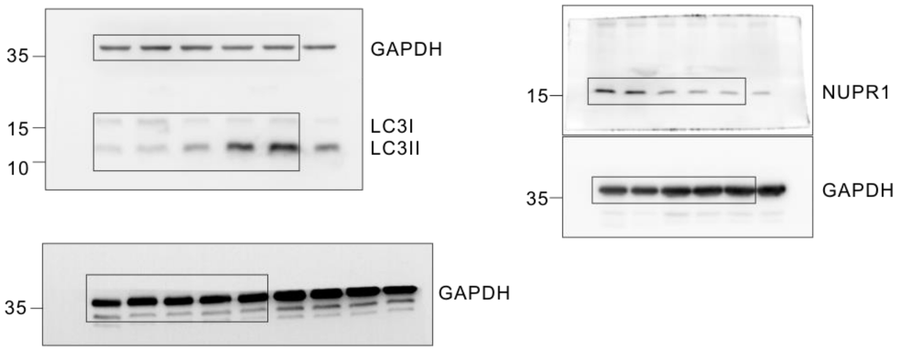

Fig. 1 f

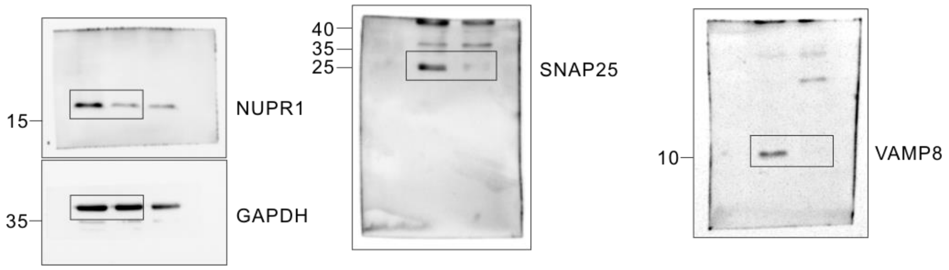

Fig. 1 g

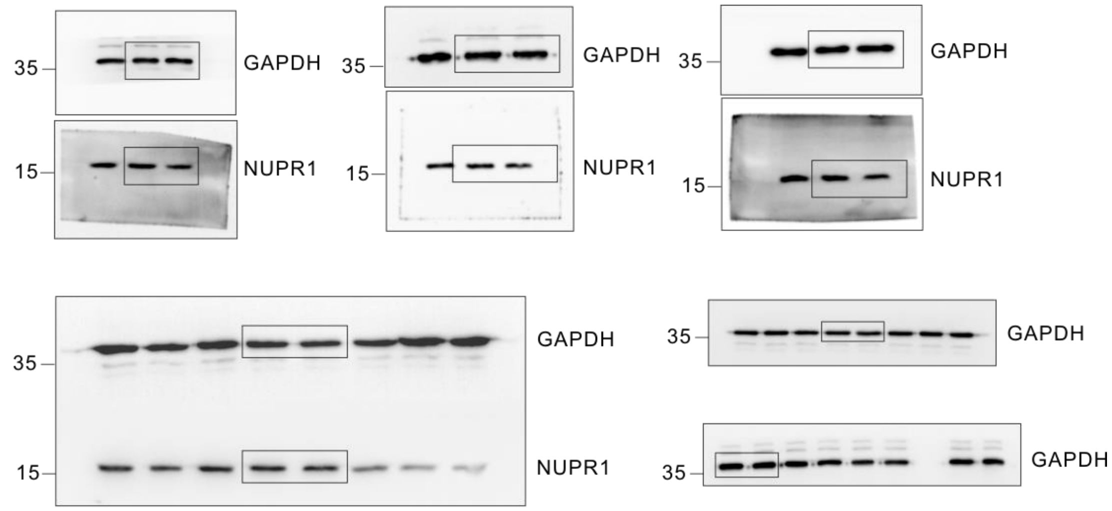

Fig. 2 c

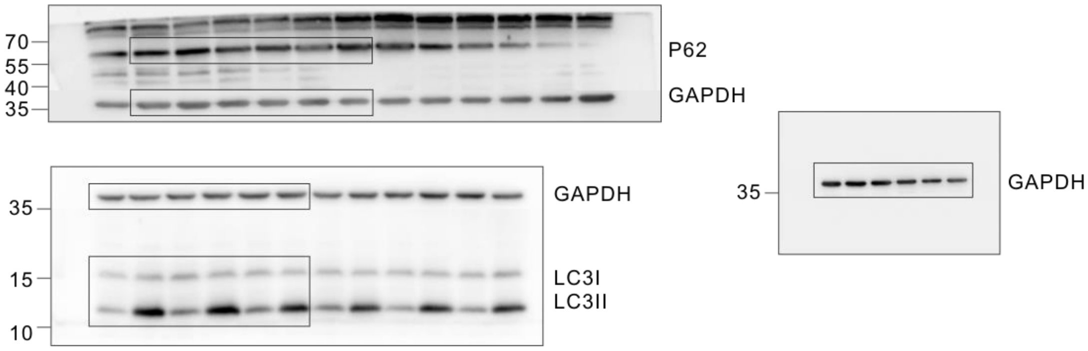

Fig. 4 c

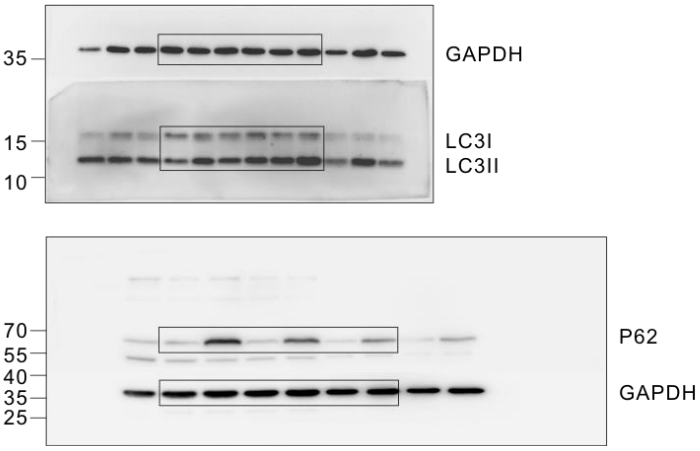

Supplementary Figure 28. Uncropped scans of western blots included in main figures.

Supplementary Table 1. shRNA sequences[3-6] used in this study.

shRNA	Sequence(5'-3')
shATG5#1	GCTTCGAGATGTGTGGTTTGG
shATG5#2	GCAACTCTGGATGGGATT
shATG7#1	GCCTGCTGAGGAGCTCTCCAT
shATG7#2	CAGTTTGGCACAATCAATA
shScramble	TTCTCCGAACGTGTCACGT

Supplementary Table 2. Blood test results for treated mouse Nude mice were administered intravenously (i.v.) with control normal saline (NS, 200 μL, n = 3), NDs (5 mg kg^{-1}, n = 9) or CQ (10 mg kg^{-1}, n = 9).

Biochemistry

Item	Control	CQ			NDs		
		6 h	12 h	24 h	6 h	12 h	24 h
AST (U L^{-1})	112.33 ± 10.21	160.00 ± 14.51*	148.33 ± 26.04	123.00 ± 17.96	128.67 ± 5.31	129.33 ± 25.98	107.67 ± 4.78
ALT (U L^{-1})	45.00 ± 4.97	49.67 ± 6.02	41.33 ± 0.47	39.00 ± 3.74	41.33 ± 3.40	41.67 ± 5.25	36.67 ± 2.36
ALP (U L^{-1})	326.33 ± 6.94	313.00 ± 27.31	316.67 ± 8.18	325.00 ± 16.99	305.33 ± 11.03	323.67 ± 34.24	335.33 ± 20.68
TP (g L^{-1})	54.77 ± 4.98	50.27 ± 1.11	51.30 ± 0.36	53.27 ± 3.81	52.37 ± 0.68	52.63 ± 1.80	52.00 ± 0.94
ALB (g L^{-1})	30.63 ± 0.31	27.50 ± 0.73**	28.90 ± 0.86	29.70 ± 1.43	29.60 ± 0.91	30.27 ± 0.69	29.50 ± 0.50
BUN (mmol L^{-1})	10.90 ± 0.62	10.80 ± 1.71	11.07 ± 1.53	9.50 ± 1.34	10.47 ± 1.14	10.40 ± 0.71	9.83 ± 0.54
CRE (μmol L^{-1})	23.00 ± 3.56	34.33 ± 1.70*	24.00 ± 1.41	22.00 ± 2.83	26.67 ± 0.47	20.33 ± 3.09	26.00 ± 3.56
GLU (mmol L^{-1})	0.81 ± 0.13	0.12 ± 0.04**	0.07 ± 0.01**	0.56 ± 0.71	0.69 ± 0.07	0.81 ± 0.56	0.71 ± 0.55
UA (μmol L^{-1})	173.33 ± 5.44	194.33 ± 12.66	197.67 ± 13.70	161.67 ± 32.67	186.67 ± 28.80	190.33 ± 16.60	163.67 ± 14.82
TG (mmol L^{-1})	0.14 ± 0.04	0.20 ± 0.03	0.21 ± 0.09	0.22 ± 0.11	0.17 ± 0.13	0.15 ± 0.03	0.18 ± 0.03
CHO (mmol L^{-1})	2.23 ± 0.12	2.63 ± 0.21	2.53 ± 0.25	2.60 ± 0.29	2.70 ± 0.29	2.47 ± 0.05	2.60 ± 0.16
CPK (U L^{-1})	743.33 ± 203.07	1410.00 ± 116.31*	1253.67 ± 354.74	918.00 ± 196.58	827.33 ± 78.76	770.67 ± 82.14	701.67 ± 66.20
LDH (U L^{-1})	793.00 ± 131.74	1159.33 ± 237.49	1448.33 ± 183.26*	1068.67 ± 116.76	876.67 ± 62.96	854.67 ± 54.96	801.00 ± 78.50

Haematology

Item	Control	CQ			NDs		
		6 h	12 h	24 h	6 h	12 h	24 h
WBS (10^9 L^{-1})	3.10 ± 0.93	4.60 ± 0.99	4.27 ± 0.63	2.90 ± 0.62	4.13 ± 0.97	3.50 ± 0.57	2.90 ± 0.80
RBC (10^9 L^{-1})	9.53 ± 0.20	10.25 ± 0.75	9.97 ± 0.07*	10.02 ± 0.25	10.10 ± 0.34	10.04 ± 0.41	9.88 ± 0.14
HGB (g L^{-1})	151.67 ± 2.87	162.33 ± 11.15	158.67 ± 2.62	160.00 ± 5.66	159.67 ± 5.91	160.33 ± 6.02	159.33 ± 3.68
HCT (%)	47.97 ± 0.42	49.90 ± 2.72	50.50 ± 0.54**	50.43 ± 1.46	51.20 ± 1.61	49.37 ± 0.71	49.07 ± 1.20
MCV (fL)	50.37 ± 1.45	48.77 ± 1.62	50.70 ± 0.22	50.37 ± 0.29	50.77 ± 1.39	51.57 ± 0.79	50.60 ± 0.16
MCH (pg)	15.93 ± 0.17	15.87 ± 0.21	15.90 ± 0.14	15.93 ± 0.21	15.83 ± 0.31	15.97 ± 0.05	16.13 ± 0.24
MCHC (g L^{-1})	316.33 ± 8.58	325.33 ± 6.60	314.00 ± 2.16	317.00 ± 2.16	311.67 ± 4.03	309.67 ± 3.30	319.00 ± 5.10
PLT (10^9 L^{-1})	1088.67 ± 131.45	1077.67 ± 219.08	1109.67 ± 178.93	1180.67 ± 28.00	903.33 ± 103.24	1176.33 ± 122.13	916.67 ± 110.29
LYMPH (%)	78.53 ± 2.78	66.33 ± 8.80	73.80 ± 9.51	55.23 ± 4.48**	75.57 ± 12.55	71.70 ± 13.54	82.10 ± 6.08
EO (%)	0.00 ± 0.00	0.07 ± 0.09	0.10 ± 0.14	0.10 ± 0.14	0.00 ± 0.00	0.00 ± 0.00	0.17 ± 0.24
BASO (%)	19.07 ± 1.38	17.07 ± 10.62	13.57 ± 0.87**	23.73 ± 9.53	16.60 ± 3.47	14.77 ± 2.80	18.80 ± 5.12
MONO (%)	2.33 ± 0.21	23.83 ± 3.60*	17.07 ± 2.98*	7.60 ± 5.87	2.87 ± 1.16	1.10 ± 0.79	1.43 ± 0.65
NEUT (%)	0.93 ± 0.38	2.43 ± 1.60	3.63 ± 0.87*	3.33 ± 0.76*	1.80 ± 0.86	1.30 ± 0.70	0.87 ± 0.31
RDW-CV (%CV)	24.80 ± 0.99	24.40 ± 0.85	25.77 ± 0.66	26.23 ± 0.54	26.70 ± 1.20	24.33 ± 0.85	25.53 ± 0.58
MPV (fL)	7.33 ± 0.12	7.60 ± 0.51	7.43 ± 0.24	7.23 ± 0.19	7.73 ± 0.31	7.37 ± 0.17	7.53 ± 0.33

Supplementary Table 3. A certain symptom number in tumor bearing mice after treatment.

		Symptom		
		Jaundice */#	Ascites */#	Liver cirrhosis */&
Treatments	NS	7/22	4/22	3/6
	NDs	8/22	3/22	3/6
	ATO 0.75 mg kg^{-1}	6/20	2/22	2/6
	NDs+ATO 0.75 mg kg^{-1}	3/20	0/20	0/9
	ATO 1.5 mg kg^{-1}	6/20	2/20	2/9
	NDs+ATO 1.5 mg kg^{-1}	1/20	0/20	0/10
	NDs+ATO 3 mg kg^{-1}	5/20	1/20	1/9
	NDs+ATO 4 mg kg^{-1}	5/20	1/20	1/7

*The number of mice with a certain symptom
The total number of mice in each group
& The total number of mice in each group for liver cirrhosis observation

Supplementary Table 4. Coefficients of major organs in nude mice after various treatments as indicated in Fig. S15. *$P < 0.05$, significantly different from NS. &$P < 0.05$, &&$P < 0.01$, &&&$P < 0.001$, significantly different from normal.

		Coefficiences of major organs(mg g^{-1})				
		Heart	Liver	Spleen	Lung	Kidney
	Normal	6.73±0.80	61.52±3.00	5.94±1.68	6.80±0.49	21.79±1.30
Treatments	NS	6.75±0.86	59.67±8.30	12.41±3.2$^{\&\&\&}$	8.22±1.71	20.72±2.0
	NDs	6.87±1.64	60.54±9.60	12.75±4.17$^{\&\&}$	7.55±0.82	18.94±1.95
	ATO 0.75 mg kg^{-1}	6.62±0.79	61.70±11.92	10.42±2.33$^{\&}$	7.47±0.79	19.66±1.70
	NDs+ATO 0.75 mg kg^{-1}	6.28±0.98	59.85±9.76	8.46±1.09$^{\&*}$	7.72±0.90	19.07±1.08
	ATO 1.5 mg kg^{-1}	6.47±0.78	60.41±11.9	9.91±2.16$^{\&\&}$	8.29±1.09	19.83±1.74
	NDs+ATO 1.5 mg kg^{-1}	6.87±1.18	59.23±6.79	7.81±1.53*	7.57±1.64	19.91±1.87
	NDs+ATO 3 mg kg^{-1}	6.57±1.12	56.12±9.88	9.57±2.68$^{\&\&}$	7.39±0.93	19.87±1.72
	NDs+ATO 4 mg kg^{-1}	6.43±0.87	67.48±10.66	9.02±2.58$^{\&}$	8.01±1.18	21.46±1.51

Reference：

1. Mu Y, Yan X, Li D, Zhao D, Wang L, Wang X, Gao D, Yang J, Zhang H, Li Y, Sun Y, Wei Y, Zhang Z, Chang X, Yao Z, Tian S, Zhang K, Terada LS, Ma Z, Liu Z. NUPR1 maintains autolysosomal efflux by activating SNAP25 transcription in cancer cells. *Autophagy*, 1-17 (2017).

2. Fullgrabe J, Lynch-Day MA, Heldring N, Li WB, Struijk RB, Ma Q, Hermanson O, Rosenfeld MG, Klionsky DJ, Joseph B. The histone H4 lysine 16 acetyltransferase hMOF regulates the outcome of autophagy. *Nature* **500**, 468-472 (2013).

3. Xiong L, Liu ZP, Ouyang GQ, Lin LW, Huang H, Kang HX, Chen W, Miao XY, Wen Y. Autophagy inhibition enhances photocytotoxicity of Photosan-II in human colorectal cancer cells. *Oncotarget* **8**, 6419-6432 (2017).

4. Huang SS, Ding DF, Chen S, Dong CL, Ye XL, Yuan YG, Feng YM, You N, Xu JR, Miao H, You Q, Lu X, Lu YB. Resveratrol protects podocytes against apoptosis via stimulation of autophagy in a mouse model of diabetic nephropathy. *Sci Rep-Uk* **7**, 45692 (2017).

5. Xue F, Hu L, Ge RL, Yang LX, Liu K, Li YY, Sun YF, Wang K. Autophagy-deficiency in hepatic progenitor cells leads to the defects of stemness and enhances susceptibility to neoplastic transformation. *Cancer Lett* **371**, 38-47 (2016).

6. Pyo JO, Nah J, Kim HJ, Lee HJ, Heo J, Lee H, Jung YK. Compensatory activation of ERK1/2 in Atg5-deficient mouse embryo fibroblasts suppresses oxidative stress-induced cell death. *Autophagy* **4**, 315-321 (2008).
